# Why do eukaryotic proteins contain more intrinsically disordered regions?

**DOI:** 10.1371/journal.pcbi.1007186

**Published:** 2019-07-22

**Authors:** Walter Basile, Marco Salvatore, Claudio Bassot, Arne Elofsson

**Affiliations:** 1 Science for Life Laboratory, Stockholm University, Solna, Sweden; 2 Department of Biochemistry and Biophysics, Stockholm University, Stockholm, Sweden; 3 Swedish e-Science Research Center (SeRC), Stockholm, Sweden; University of Texas at Austin, UNITED STATES

## Abstract

Intrinsic disorder is more abundant in eukaryotic than prokaryotic proteins. Methods predicting intrinsic disorder are based on the amino acid sequence of a protein. Therefore, there must exist an underlying difference in the sequences between eukaryotic and prokaryotic proteins causing the (predicted) difference in intrinsic disorder. By comparing proteins, from complete eukaryotic and prokaryotic proteomes, we show that the difference in intrinsic disorder emerges from the linker regions connecting Pfam domains. Eukaryotic proteins have more extended linker regions, and in addition, the eukaryotic linkers are significantly more disordered, 38% vs. 12-16% disordered residues. Next, we examined the underlying reason for the increase in disorder in eukaryotic linkers, and we found that the changes in abundance of only three amino acids cause the increase. Eukaryotic proteins contain 8.6% serine; while prokaryotic proteins have 6.5%, eukaryotic proteins also contain 5.4% proline and 5.3% isoleucine compared with 4.0% proline and ≈ 7.5% isoleucine in the prokaryotes. All these three differences contribute to the increased disorder in eukaryotic proteins. It is tempting to speculate that the increase in serine frequencies in eukaryotes is related to regulation by kinases, but direct evidence for this is lacking. The differences are observed in all phyla, protein families, structural regions and type of protein but are most pronounced in disordered and linker regions. The observation that differences in the abundance of three amino acids cause the difference in disorder between eukaryotic and prokaryotic proteins raises the question: Are amino acid frequencies different in eukaryotic linkers because the linkers are more disordered or do the differences cause the increased disorder?

## Author Summary

Intrinsic disorder is essential for various functions in eukaryotic cells and is a signature of eukaryotic proteins. Here, we try to understand the origin of the difference in disorder between eukaryotic and prokaryotic proteins. We show that eukaryotic proteins contain more extended linker regions and that these linker regions are significantly more disordered. Further, we show, for the first time, that the difference in disorder originates from a systematic difference in amino acid frequencies between eukaryotic and prokaryotic proteins. Three amino acids contribute to the difference in disorder; serine and proline are more abundant in eukaryotic linkers, while isoleucine is less frequent. These shifts in frequencies are observed in all phyla, protein families, structural regions and type of protein but are most pronounced in disordered and linker regions. It is tempting to speculate that the increase in serine frequencies in eukaryotes is related to regulation by kinases, but direct evidence for this is lacking. Anyhow the widespread of the shifts in abundance indicates that the differences are ancient and caused be some yet not fully understood selective difference acting on eukaryotic and prokaryotic proteins.

## Introduction

Eukaryotic cells are more complex than prokaryotic cells, and therefore, have an increased need for regulation. They also contain organelles, have more complex genes and a more advanced chaperonin system enabling the folding of longer proteins [1]. In response to the increased complexity, eukaryotic proteomes have evolved to differ significantly from prokaryotic proteomes. The most notable differences are that; (i) eukaryotic proteins are longer [2–5], (ii) multi-domain proteins are more abundant in eukaryotes [6–8], (iii) domain repeats are frequent in multicellular organisms [9], and (iv) eukaryotic proteins have a higher fraction of disordered residues [10].

The increased length of eukaryotic proteins is, at least partly, a consequence of them containing more domains [11]. With more multi-domain proteins, it follows that eukaryotic proteins have more linker regions—connecting the domains [12]. Further, the increased number of domain repeats appears to be a unique feature of multicellular organisms [9]. These repeats have been proposed to provide eukaryotes with an additional source of variability to compensate for low generation rates [13] and are important for signalling.

The origin of the increase in intrinsic disorder in eukaryotic proteins is less well understood. Intrinsic disorder is frequent in all eukaryotic phyla, and even among viral proteins [14]. In earlier studies, about 10% of the residues in prokaryotes are predicted to be disordered compared with 30% in eukaryotes [15–18]. Disordered regions are over-represented in regulatory proteins [19], providing a possible explanation for the increase of intrinsic disorder in eukaryotes.

Ahrens et al. proposed that the increased intrinsic disorder in eukaryotic is a result of lower selective pressure due to the smaller effective population size in eukaryotes [15]. The observation that ancient eukaryotic genes are less disordered than young or random genes [20] supports this. However, a large number of functionally important intrinsically disordered regions have been described [21, 22]. Functions associated with disordered regions include; to present short linear motifs that are important for binding [19] and to enable post-translational modification that preferentially occurs in intrinsically disordered regions [23, 24]. Likely, at least some of the intrinsically disordered regions in eukaryotic proteins are functionally important.

The vast majority of studies of intrinsic disorder are based on predictions [25] and although the best predictors use multiple sequence alignments [26], even simple predictors that only use the amino acid sequence identify the difference between eukaryotes and prokaryotes [27]. The average “disorder propensity”, as measured by the TOP-IDP scale [28], is also significantly higher for eukaryotic proteins than for prokaryotic proteins. Polar and charged amino acids, together with proline, are the most disorder-promoting residues. Thus, proteins with a higher fraction of these residues are (predicted to be) more disordered. Therefore, there should be an increase in the abundance of these amino acids in eukaryotic proteins or a decrease of the order promoting residues. However, to the best of our knowledge, shifts of amino acid frequencies between eukaryotic and prokaryotic proteins have not earlier been used to analyse the difference in intrinsic disorder.

Over evolutionary times there exist many possibilities for amino acids to change in a protein family without the loss of function [29]. Most protein families contain members that have less than 20% sequence identities [30]; i.e. for most proteins, it is possible to replace more than 80% of the residues and still maintain its function. Further, protein design experiments have shown that it is possible to design functional proteins with a limited [31], or biased [32], set of amino acids. Therefore, an organism should be able to adapt its amino acid frequencies if an advantage to do so exists.

Multiple factors can affect systematic shifts of amino acids frequencies, and one of the most notable is the GC content of the genome. Amino acids encoded by high GC codons are enriched in high GC genomes and vice versa. This trend is particularly strong among recently created genes but also exists for ancient genes [20]. It has been shown that amino acids with codons enriched in GC are disorder-promoting [33], explaining why *de novo* created proteins in yeast (low GC) appear to be ordered while in Drosophila (high GC) such proteins are predicted to be disordered [20].

The general trend of amino acid gains and losses has also been studied before, and it has been proposed that the amino acids (except serine) that appeared to increase in frequency recently were not incorporated in the first genetic code [34]. However, the statistical methodology used in that study has been questioned [35]. Further, it has been observed that the frequency of tyrosine has decreased in Metazoans compared to yeast [36], and histidine and serine frequencies increase from high-temperature thermophiles to prokaryotic mesophiles and further to eukaryotes while valine shows the opposite trend [37]. Finally, a trend of increasing polar amino acids in eukaryotes has been reported [38]. Some of these changes can contribute to the increased disorder in eukaryotes, but until now, studies of intrinsic disorder have not taken shifts of amino acid frequencies into account.

In this study, we try to identify the molecular properties that underlie the difference in intrinsic disorder between eukaryotes and prokaryotes. First, we show that the difference in disorder can primarily be attributed to that linker regions in eukaryotes are, not only more abundant but also more disordered. Next, we show that differences in serine, proline, and isoleucine frequencies can explain the difference in intrinsic disorder between eukaryotic and prokaryotic linkers.

## Materials and methods

### Datasets

The dataset used in this study originates from the complete bacterial, archaeal and eukaryotic proteomes in UniProt [39] as of December 2017. However, differences in GC composition complicate the comparison of amino acid distributions as the frequency of some amino acids is strongly dependent on the GC content of the genome, S1 Fig. In the prokaryotic kingdoms, there exist a significant fraction of genomes with high GC content, S2 Fig. We tried several methods to compensate for differences in amino acid frequencies caused by the differences in GC. One possibility is to use an ANOVA test, S1 Table. The general conclusions are similar using any of these methods, but if GC is completely ignored significant differences can be missed.

After several tries, we do believe that the easiest way to compensate for GC is to ignore all genomes with extreme GC content. In addition to the simplicity, this removal also makes it possible to compare trends within protein families without compensating for the GC content. Therefore, we excluded all genomes with a GC content of more than 60% or less than 20%. The resulting set of genomes have a similar GC content in all three kingdoms, and the average GC is 43-44% with a standard deviation of 8%, S2b Fig. All genomes from Mycoplasma, Spiroplasma, Ureaplasma, and Mesoplasma were also ignored as they have another codon usage—which influences the expected amino acid frequencies. The final dataset contains 26,274,724 protein sequences from 6,373 genomes, divided into 4,905 bacterial, 308 archaeal, and 975 eukaryotic.

### Protein regions

**Fig 1:**
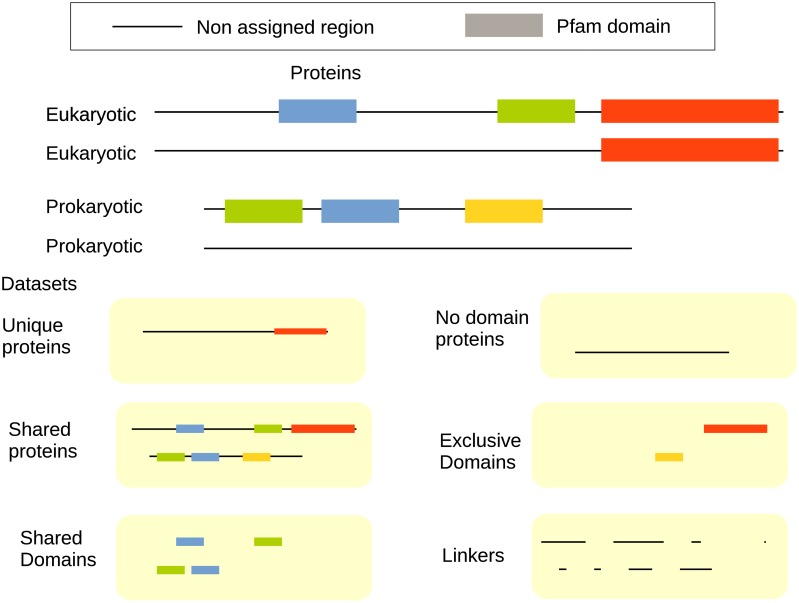
Division of proteins into six subsets: First all proteins are divided into three groups: “kingdom specific proteins” that only contain domains unique to one of the kingdoms, “no domain proteins” without any domains and “shared proteins” that contains at least one of the “shared domains”. The last group is then further divided into three regions: “shared domains”, “specific domains”, and “linkers”

Different numbers of proteins of a particular type or differences within proteins of the same type can cause differences at the proteome level. To distinguish these scenarios, we divide the complete proteomes into subsets using Pfam [40, 41]. First, we identified 4,165 shared Pfam domains that are present in at least ten eukaryotes and ten prokaryotes, and where none of the kingdoms makes up of more than 99.9% of all the members. 1,764 of these domains are present in all three kingdoms. We define a set of “shared proteins” as all proteins that contain at least one of these “shared domains”. Proteins that only contain Pfam domains that are unique to one of the kingdoms are referred to as (kingdom) “specific proteins”, and proteins without any Pfam domains are called “no domain” proteins, see Fig 1.

Also, within one group of proteins, proteome-wide differences might be caused by the abundance of different regions or differences within similar regions. Therefore, we divided the “shared proteins” further into regions, see Fig 1. Regions corresponding to any of the 4,165 Pfam domains, that exist both in prokaryotes and eukaryotes, are called “shared domains”, while regions assigned to any other Pfam domain are “specific domains”, and all regions that are not assigned to a Pfam domain are classified as “linker regions”. The linker regions plus the no-domain proteins should be similar but not identical to the dark proteome [42].

For each of these six groups, we analysed length, disorder, amino acid frequencies, and other properties independently. As shown in Fig 1, a protein can contain zero, one or multiple regions of a particular type. Therefore, if a protein contains two “shared domains” the length of shared domains in that protein is the sum of the length of both the domains. The processed datasets, as well as all scripts, are available from https://figshare.com/articles/Dataset_for_paper/7478381.

### Disorder prediction

For each protein, we estimated the intrinsic disorder using two tools: IUPred [27] and TOP-IDP [28]. IUPred exploits the idea that in disordered regions, amino acid residues form less energetically favourable contacts than residues in ordered regions. IUPred does not rely on any external information besides the amino acid sequence and is therefore extremely fast and suitable to predict disorder for large data sets. We used the recommended cut-off and assigned a residue to be disordered if its IUPred value is higher than 0.4 [43, 44]. We report the results using IUPred long disordered predictions. Using the short version of IUPred or a different cut-off gives almost identical results, S2–S4 Tables. We also calculate the average disorder propensity using the TOP-IDP scale [28] for each region.

### Statistical analysis

Properties, including length, amino acid type and disorder were analysed independently for each protein region, as described in Fig 1. Comparisons were performed between regions of different types and between kingdoms. Statistical significances were calculated using Students T-tests, but the numerous data points make even small differences statistically significant. For instance, the predicted number of disordered residues among the shared domains is small (21.3 in bacteria vs. 27.1 in eukaryotes), S2 and S3 Tables, but significant (*P* < 1.3 * 10^−8^). For many other comparisons, the P-values are smaller than 10^−200^. Therefore, we do not believe it is of relevance to report each P-value for all comparisons. Instead, we have just included the standard errors in relevant figures and S2–S4 Tables.

## Results and discussion

First, we compare the average length and disorder content for proteins in the different kingdoms of life. In total, the proteomes contain 26 million proteins. About half (14 million) of the proteins belong to the group of “shared proteins”, i.e. they contain at least one Pfam domain that exists in both prokaryotes and eukaryotes. These proteins can, therefore, be assumed to be the most ancient. The next group consists of the 4 million “kingdom specific proteins”, which only contain Pfam domains that are unique to one of the kingdoms. These proteins are more likely to be more recent innovations and could perform functions specific to properties unique to one of the kingdoms. Finally, we have 8.3 million proteins without any annotated Pfam domain, most likely, these are the youngest proteins, but this group could also incorporate some fast-evolving proteins. Next, the “shared proteins” are studied in more detail by dividing these proteins into three regions: regions with a “shared domain”, regions with a “specific domain” and regions without domain annotations, i.e. “linker regions”, see Fig 1.

### Eukaryotic proteins have more extended linker regions

**Fig 2:**
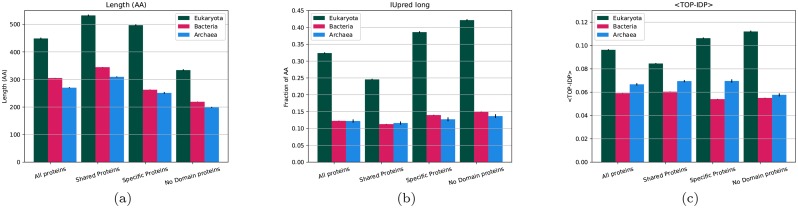
Average properties of proteins from different kingdoms; (a) average length, (b) fraction of residues predicted to be disordered by IUPred and (c) average TOP-IDP scores. Error bars represent the standard error for each property.

As shown before [5, 8, 45], eukaryotic proteins are on average longer than prokaryotic proteins see Fig 2 and S2–S4 Tables. The group of proteins with “shared domains” is longer than proteins with only “specific domains”, and the proteins without domains are even shorter. However, in all three groups, eukaryotic proteins are significantly longer than the prokaryotic proteins.

We have, in an earlier study, contributed the difference in length to that eukaryotic proteomes contain more multi-domain proteins [5]. In that study, we assumed that long linker regions contained missed domains, and this contributed to the assumption that the increase in multi-domain proteins was a driver for the difference in length between eukaryotic and prokaryotic proteins. However, given the insights from studies of disordered regions [12] and the dark proteome [42], it is now clear that long linker regions do not necessarily contain unassigned domains. Therefore, we do not assign domains to long unassigned regions.

**Fig 3:**
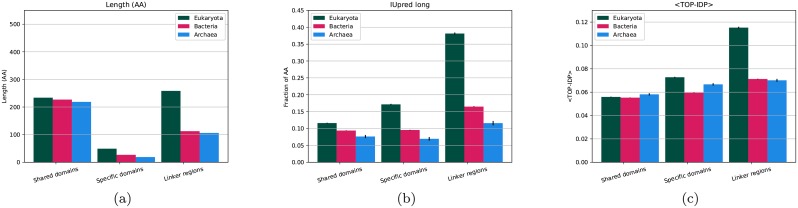
Average properties of proteins regions from different kingdoms; (a) average length, (b) fraction of residues predicted to be disordered by IUPred and (c) average TOP-IDP scores. Error bars represent the standard error for each property.

To understand the origin of the difference in length between eukaryotic and prokaryotic proteins, we choose to study the shared proteins in more details. Among the 14 million proteins with “shared domains” the average length of the eukaryotic proteins is 532 vs. 345 for bacterial protein and 309 for proteins in Archaea. The number of residues in “shared domains” is roughly equal in the three kingdoms, 218 to 233, and the average number of residues assigned to “kingdom specific domains” is, although higher in eukaryotes, quite low (27 in bacteria, 19 in Archaea, and 49 in eukaryotes), see Fig 3. In contrast, the number of residues in “linker regions” differs significantly between the kingdoms, in eukaryotes, 48% of all residues are assigned to “linker regions”, compared to only 31% in prokaryotes, S2–S4 Tables. Thus, the length of “linker regions” comprises > 80% of the length difference between eukaryotic and prokaryotic proteins.

Eukaryotic proteins have more residues assigned to linker regions. Linkers can be located at one of the termini or between two domains. In all three kingdoms, each of the termini contains roughly 40% of the linker residues, and linkers between domains (central) the remaining 20%, S2–S4 Tables. Independent on location, linkers are more than twice as long in eukaryotes than in prokaryotes.

To understand how the linkers differ between eukaryotes and prokaryotes, it is necessary also to consider differences between eukaryotic and prokaryotic domains. Many Pfam domains only cover the central most conserved core of a domain and not variable regions at the termini [46]. Eukaryotic domains are known to show increased variability, possibly contributing to the extended linker regions [47]. Therefore, it is not impossible that extensions of existing domains cause some of the increased linker lengths in eukaryotes. However, we do believe that these additional residues should not be significantly more ordered than other residues within the domains. Therefore, variations within domains should not be the principal cause for the increased disorder in eukaryotic proteins.

### Eukaryotic linkers are more disordered

Next, we studied the disorder in the different groups of proteins. All three groups of eukaryotic proteins are more disordered than prokaryotic ones, see Fig 2. In agreement with earlier studies [15, 18, 48, 49], 12% of the residues in prokaryotes are predicted to be disordered compared with 32% in eukaryotes, S2–S4 Tables. Proteins that are unique to eukaryotes are more disordered than those that contain “shared domains”, and eukaryotic proteins without any Pfam domains are the most disordered with 42% disordered residues. The observation that proteins unique to eukaryotes have increased disorder supports the earlier observations that young eukaryotic proteins are more disordered than older proteins [20]. For prokaryotic proteins, the disorder content in all three groups of proteins is lower (11-15%).

To understand the origin of the difference in disorder better, we studied disorder in the different regions of the proteins that contain a “shared domain”. First, it can be seen that eukaryotic “specific domains” are more disordered than all other types of prokaryotic or eukaryotic domains, 17% vs. 8-12%, S2–S4 Tables. However, the most significant difference is that eukaryotic “linker regions” are much more disordered (38%) than prokaryotic “linker regions” (12-16%), see Fig 3. The difference in disorder can therefore not only be contributed to that “linker regions” are more abundant in eukaryotic proteins, but also to that eukaryotic linkers contain a higher fraction of disordered residues.

### Eukaryotic proteins have, on average, 145 disordered residues

**Fig 4:**
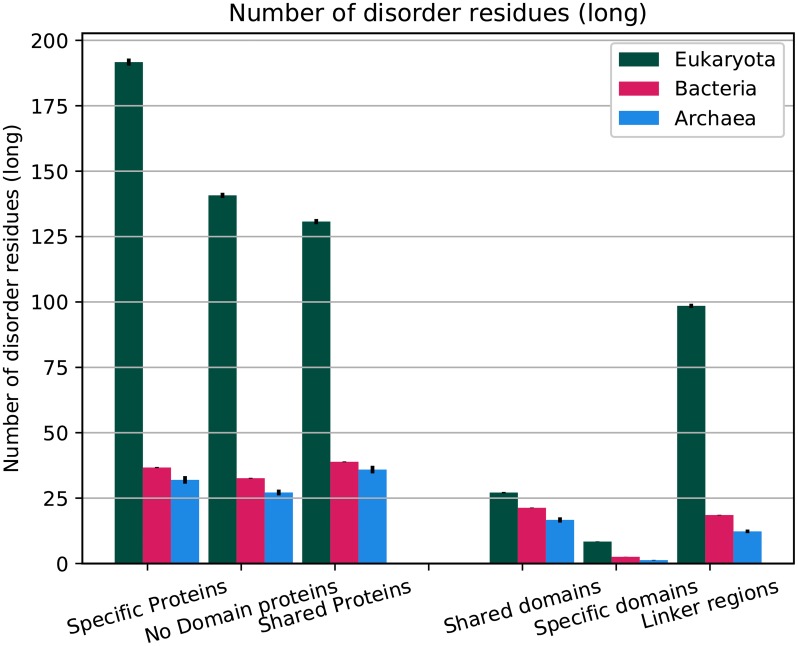
Average number of residues predicted to be disordered in different protein groups and regions. Error bars represent the standard error for each property.

Eukaryotic and prokaryotic proteins differ both in lengths of different regions and in disorder content. Therefore, it might be of interest to describe an average eukaryotic and prokaryotic protein. The average eukaryotic protein is 450 residues long and contains 32% disordered residues, while an average prokaryotic protein is 300 residues long and contains 12% disordered residues, which infers that the average eukaryotic protein contains 145 disordered residues compared with 32-37 for the prokaryotic proteins, see Fig 4. Next, eukaryotic proteins have much longer linker regions with 258 vs ≈ 110 residues in prokaryotes, and the eukaryotic linker regions are more disordered, see Fig 3. 100 of the disordered residues in eukaryotic proteins are located in the linker regions, while prokaryotic linker regions only contain 12-18 disordered residues, S2–S4 Tables. The number of disordered residues within the domains is higher in eukaryotic proteins, 36 vs 17-24. Anyhow, this demonstrates that the increase in disorder is dominated by the increase in disorder within the linkers.

### AA frequencies in eukaryotic linkers are unique

Above, we show that eukaryotic proteins are more disordered than prokaryotic proteins because their “linker regions” are both longer and more disordered. However, (predicted) intrinsic disorder is primarily caused by differences in amino acid frequencies. Therefore, we studied the difference in amino acid frequencies between eukaryotic and prokaryotic proteins.

**Fig 5:**
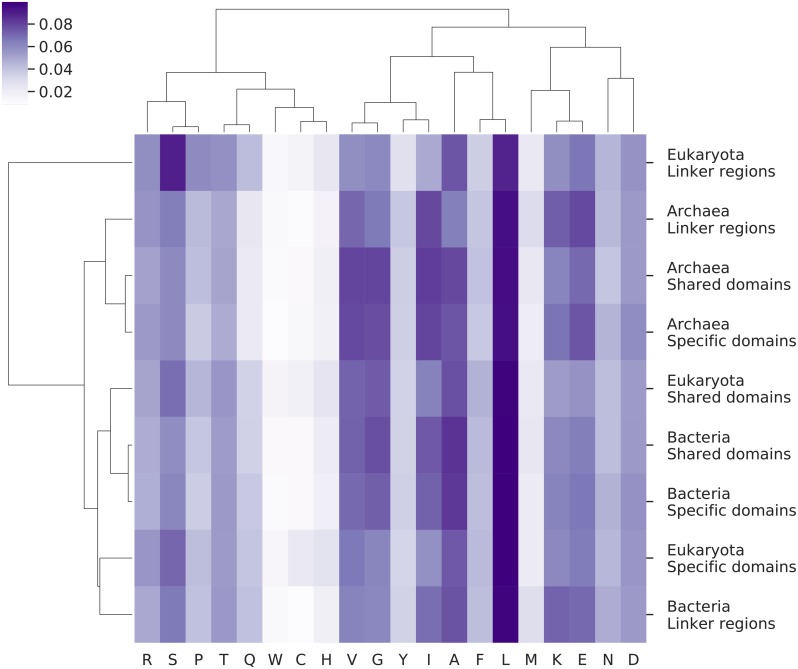
Heat map showing the similarity of amino acid frequency profiles in different regions as measured by the Pearson correlation coefficient. The colour of each cell represents the frequency of each amino acid in that region, according to the reference colour bar.

One way to compare properties of different regions is to compare the amino acid distributions in the entire regions and then cluster the regions, see Fig 5. In the heat map, the most substantial difference between regions is that the amino acid frequencies of eukaryotic linkers are distinct from all other regions. It can also be observed that all regions in Archaea cluster together, while the eukaryotic domains and all bacterial regions form the third cluster. However, this difference is much smaller.

**Fig 6:**
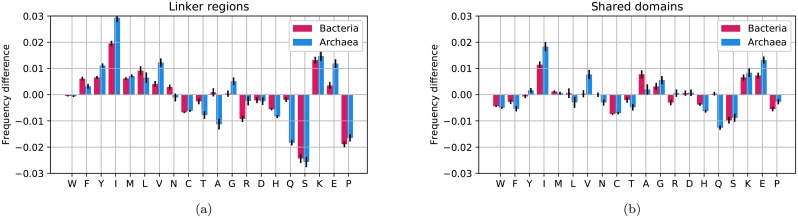
Differences in amino acid frequency between eukaryotes and prokaryotes (red for bacteria, blue for Archaea) for “linker regions” (a) and “shared domains” (b). All comparisons are made using the eukaryotic frequencies as a baseline, i.e. if an amino acid (such as serine) is more abundant in eukaryotes; the shift is downwards as this amino acid is less frequent in prokaryotes. Error bars represent the standard error for each amino acid.

To understand what causes the eukaryotic linkers to have unique amino acid distributions, we compared the amino acid frequencies between eukaryotic and prokaryotic regions, see Fig 6 and Table 1. Here, it can be seen that there exist three amino acid, isoleucine, serine and proline, whose frequencies differ by more than 1.5% between eukaryotic “linker regions” and linkers in either of the prokaryotes. These differences are also notable in the heat map, see Fig 5. Further, the frequencies of these amino acids also differ within the shared domains, but to a smaller degree, see Fig 6b. Finally, a two-way ANOVA test shows that isoleucine, proline and serine are the amino acids with the most significant differences between the eukaryotic and bacterial proteins when including the GC content, S1 Table. It should be noted that the shifts of isoleucine and proline are small if the GC content of the genomes is ignored. However, the increase in serine frequency among eukaryotes is easy to detect, and it is a surprise to us that this has not been highlighted before.

**Table 1:** Amino acid frequencies for each protein, region and kingdom. For standard errors see supplementary S2–S4 Tables.

	Shared domains	Eukaryotes Specific domains	Linker regions	Shared domains	Bacteria Specific domains	Linker regions	Shared domains	Archaea Specific domains	Linker regions
Trp	1.5%	1.3%	1.1%	1.0%	1.0%	1.1%	1.0%	0.8%	1.1%
Phe	4.6%	4.3%	3.6%	4.3%	4.2%	4.2%	4.0%	3.8%	3.9%
Tyr	3.4%	3.3%	2.7%	3.4%	3.5%	3.4%	3.6%	3.6%	3.8%
Ile	6.3%	5.8%	4.9%	7.5%	7.2%	6.9%	8.1%	7.9%	7.8%
Met	2.3%	2.1%	2.2%	2.4%	2.1%	2.9%	2.3%	2.0%	3.0%
Leu	9.8%	9.8%	8.8%	9.8%	9.9%	9.7%	9.5%	9.5%	9.5%
Val	7.1%	6.5%	5.8%	7.2%	7.0%	6.2%	7.9%	7.8%	7.1%
Asn	4.2%	4.4%	4.5%	4.2%	4.6%	4.8%	3.9%	4.5%	4.4%
Cys	1.8%	2.2%	1.5%	1.0%	1.0%	0.8%	1.1%	1.2%	0.9%
Thr	5.5%	5.4%	5.7%	5.3%	5.4%	5.5%	5.0%	4.8%	4.9%
Ala	7.6%	7.4%	7.5%	8.4%	8.2%	7.5%	7.8%	7.5%	6.4%
Gly	7.3%	6.1%	6.0%	7.6%	7.2%	6.0%	7.9%	7.7%	6.5%
Arg	5.0%	5.5%	5.8%	4.7%	4.6%	4.9%	5.1%	5.4%	5.6%
Asp	5.4%	5.5%	5.6%	5.4%	5.7%	5.4%	5.4%	5.8%	5.4%
His	2.5%	2.6%	2.4%	2.1%	1.9%	1.8%	1.8%	1.7%	1.6%
Gln	3.4%	3.9%	4.2%	3.5%	3.8%	4.0%	2.2%	2.2%	2.4%
Ser	6.8%	7.1%	8.9%	5.9%	6.1%	6.5%	5.9%	5.9%	6.3%
Lys	5.3%	6.0%	5.8%	6.0%	6.3%	7.2%	6.2%	6.7%	7.3%
Glu	5.7%	6.5%	6.7%	6.4%	6.6%	7.0%	7.0%	7.5%	7.9%
Pro	4.4%	4.2%	5.9%	3.9%	3.6%	4.0%	4.2%	3.7%	4.3%

The amino acid frequency in different regions shows that not all disorder-promoting amino acids increase in frequency in eukaryotic linkers. The difference in disorder is instead caused by the shift in frequencies of only three amino acids, isoleucine, serine, and proline. All three amino acids contribute to the increased disorder in eukaryotic linkers, and if these three amino acids are ignored, there is no significant difference in disorder propensity between eukaryotes and prokaryotes, S3 Fig. However, it is not clear if the increased disorder in eukaryotic linkers is primarily a consequence of changes in amino acids frequencies, or if the need for increased disorder drives the changes in amino acid frequencies—a chicken and egg problem.

### The difference in amino acid frequencies is widespread

Eukaryotic proteomes are in general larger than prokaryotic proteomes; this is partly due to an expansion of protein families by gene duplications. For functional reasons, different protein families have different amino acid distributions. Therefore, it is possible that the differences in the amino acid frequency that we observe when studying an entire proteome are due to the different frequencies of protein families. However, to better under the origin of the amino acid frequency differences, we examined the amino acid frequency of all shared Pfam domains independently. The reason to study domains and not the linkers is that the linkers are challenging to align and differ significantly in length, while the domains are of similar length and already aligned in Pfam. Further, the serine and isoleucine differences are also present among the shared domains, see Fig 6b.

**Fig 7:**
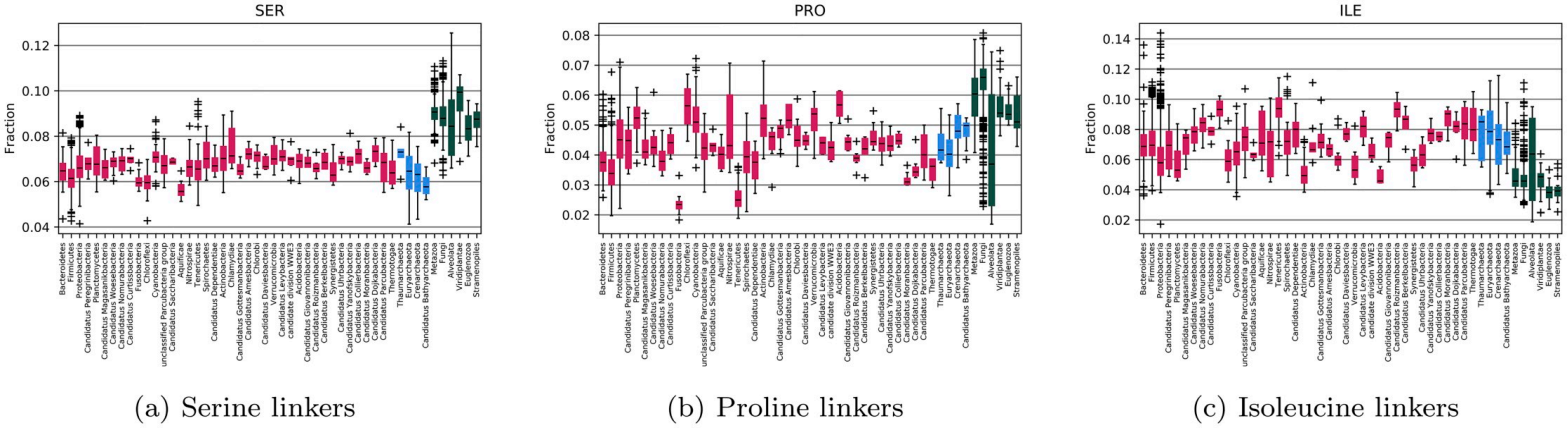
Distribution of differences in amino acids frequencies in Pfam families. Only Pfam families that contain at least 100 members in bacteria and eukaryotes are included in the comparison. The differences are measured as the shift from the observed amino acid frequency in eukaryotes. Blue bars represent Archaea and red bacteria. Differences are for (a) serine, (b) proline, and (c) isoleucine.

In Fig 7, the differences between the amino acid frequencies in the prokaryotic domains are compared with the amino acid frequencies in the corresponding eukaryotic domains. Only Pfam families with at least 100 members among both bacteria and eukaryotes are included to avoid statistical outliers (Archaea was ignored in this filtering). In 84% of the families, the eukaryotic members have more serine, in 80% fewer isoleucine and 70% more proline, i.e. the shifts in frequencies are observed in a majority of the families. We also tried to identify any trends among the families with extreme amino acid frequencies, both by examining individual families and by mapping to GO-slim terms, using pfam2go [50–52]. The GO terms with the most substantial differences in amino acid frequencies are listed in S5 Table. However, to the best of our ability, we cannot identify any particular functional subset of proteins where the difference in frequency significantly differs from the general picture. Therefore, the differences in frequencies do not appear to be caused by shifts in the frequency of some particular group of proteins. Instead, there seems to exist a systematic shift in the frequencies between eukaryotes and prokaryotes present in most protein families.

### Serine frequency is increased in all organelles

**Fig 8:**
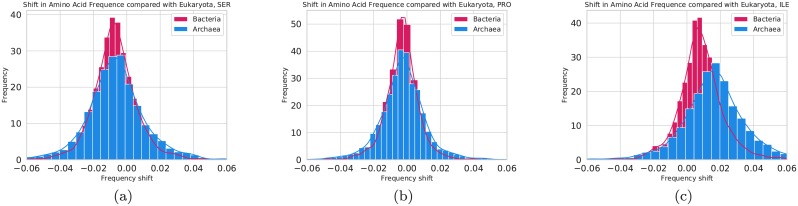
Frequency of (a) serine, (b) proline, and (c) isoleucine in linker regions in proteomes grouped by phylum. Bacterial groups are red, eukaryotic dark green, and archaeal blue.

A difference between eukaryotes and prokaryotes is that eukaryotic cells have organelles. The amino acid content of proteins in different organelles differs; therefore, it would not be implausible that the different amino acid frequencies could be affected by the compartmentalization of the eukaryotic cell. However, in all membrane and non-membrane parts of all organelles, the frequencies of serine and proline are higher in eukaryotes than in prokaryotes, see S6 Table. Further, in all but three organelles, the isoleucine frequency is lower in eukaryotes. Some bacteria within Planctomycetes, Verrucomicrobiae, and Chlamydiae have quite complex membranes, possibly indicating primitive organelles [53]. However, all these phyla have bacterial levels of serine, proline and isoleucine, see Fig 8. Therefore, the compartmentalization of the eukaryotic cell does not appear to explain the differences in amino acid frequencies between eukaryotes and prokaryotes.

### Serine is enriched in disorder regions

Within protein regions, there exist different structural elements, such as helices, sheets loops and disordered regions. Amino acids have different preferences for different structural elements. Therefore, to investigate the preferences of amino acids in different structural elements, we compared the amino acid frequencies in different structural regions within the “shared domains”. Here, we only use the Pfam families where there was at least one structure available in PDB, and we assume that the secondary structure is conserved within the entire Pfam family. The reason to use only the “shared domains” is that the structural information of the linkers is limited. Using the secondary structure annotation, available from Pfam, we then assign each residue into one out of three categories, Helix, Sheet or Coil, using the most frequent annotation in Pfam. Unassigned positions, i.e., residues corresponding to the parts of the Pfam domains that are not present in any PDB structure, we do refer to as disordered, as often done when training disorder predictors [54].

**Fig 9:**
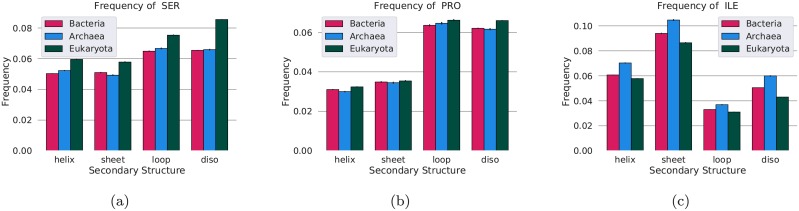
Frequency of (a) serine, (b) proline, and (c) isoleucine in different secondary structures in proteins from eukaryotes (dark green), bacteria (red) and Archaea (blue).

The amino acid frequencies in each structural region are shown in Fig 9 and S6 Fig. As expected, the serine and proline frequencies are highest in loops and disordered regions. However, when comparing amino acid frequencies between the kingdoms, it can be seen that the serine frequency is increased in all secondary structures in eukaryotes compared with prokaryotes. The most substantial difference is observed in the disordered regions (2%). For proline and isoleucine, a smaller, but still statistically significant (*P* < 10^−4^) can be observed in all secondary structure classes, i.e. he frequency differences of serine, proline and isoleucine are widespread and not unique to a particular protein element.

### What are the possible reasons for the observed frequency shifts?

What is the underlying reason for the shifts in amino acid frequencies? One possible reason for the higher fraction of serine in eukaryotic organisms is that serine, together with threonine, are targets for Ser/Thr kinases [55]. Phosphorylation of serine and threonine is one of the critical regulatory pathways in eukaryotes, but also present in Archaea [56]. Further, phosphorylation frequently occurs in intrinsically disordered sites [57]. Together this makes it intriguing to speculate that serine frequency is higher in eukaryotic linkers because of the increased need for regulation by kinases.

Ser/Thr kinases are prevalent in eukaryotes, but also exist in bacteria such as Planctomycetes [58]. The only fully sequenced genome of this phylum (*Planctomycetes bacterium GWA2_40_7*) has 6.1% serine, typical for bacteria. Further, the largest family of Ser/Thr kinases, Pfam family Stk19 (PF10494), only exists in eukaryotes and Halanaerobiales. The 2783 Halanaerobiales sequences in UniProt [39] contain 5.8% serine, also typical for a prokaryote. The bacterial levels of serine in these organisms show that the presence of Ser/Thr kinases is not necessarily causing an increase in serine frequencies.

Phosphorylation can occur at three amino acids, serine, threonine, and tyrosine. Threonine and tyrosine frequencies show no increase in eukaryotic “linker regions”, S4 Fig, even when GC is taken into account, S5 Fig. If phosphorylation by kinases is the primary reason for the serine frequency difference between eukaryotes and prokaryotes, why only serine frequency is increased? It might be due to that about 75% of the known targets for kinases are serine [59]. It might also be related to the fact that serine is a disorder-promoting residue while threonines and tyrosines are not. Although it is tempting to speculate that phosphorylation contributes to the increase of serine in eukaryotes, there exists no direct evidence that regulation by Ser/Thr kinases is the cause of the increased serine frequency.

In contrast to serine, we are not aware of any functional roles, of proline and isoleucine, that are kingdom specific, but some proline-rich structural features might be more prevalent in eukaryotes. In addition to being enriched in loops, proline is frequent in repeat proteins [60], and in particular, PPP and PPG repeats are frequent in multicellular organisms [61]. Proline repeats are also often found in disordered regions that are important for binding in eukaryotic specific proteins such as P53 [62]. Proline is also frequent in “linker regions” connecting domains [63]. As both repeats and multi-domain proteins are more frequent in eukaryotes, these factors might contribute to the increase of proline in eukaryotic proteins. However, as proline is also more frequent within the eukaryotic linker regions, this does not explain the increase in proline.

Prokaryotes (but not eukaryotes) use a specific purine-rich sequence on the 5’ side to distinguish initiator AUGs from internal ones [64]. The codons for isoleucine contain 44% Adenosine. Therefore, this could potentially contribute to the higher fraction of isoleucine in prokaryotes. However, as the frequency differences between eukaryotes and prokaryotes also exist in C-terminal regions, this cannot be the only explanation for the difference of isoleucine frequency.

### Different selective pressure in eukaryotic linkers?

**Fig 10:**
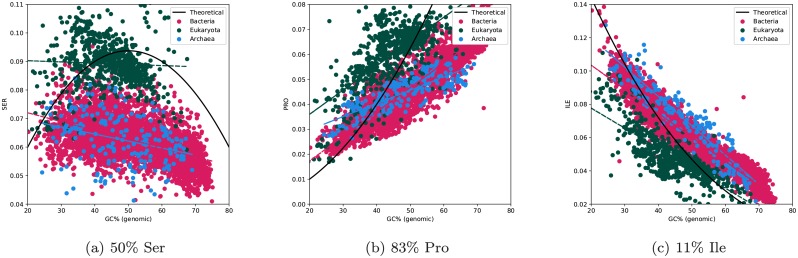
Frequency of (a) serine, (b) proline, and (c) isoleucine vs. GC of the “linker regions” in the genomes. The amino acids are sorted after the GC content of their codons. The number represents the fraction of GC among the codons. The black line represents the expected frequency from codon usage only. Here, all genomes before the filtering on GC are included for clarity.

In addition to functional reasons for the differences in frequencies, the differences could be caused by general trends in the strength of the selective pressure. Such a model would assume that there is a general preference to shift the frequency of an amino acid from what is expected by chance. Functional selection is typically considered to be the dominant force shaping proteome evolution, but secondary effects such as the cost of producing an amino acid or codon usage preferences can also affect the general trend of amino acid frequencies [65]. The population size of eukaryotes is in general smaller than for prokaryotes causing a lower selective pressure. The amount of intrinsic disorder is lower than expected by chance in both eukaryotes and prokaryotes [20]. Therefore, it is possible that the lower selective pressure could explain why eukaryotes contain more disordered residues if these residues are unfavourable [15]. However, this is not always the case as some disorder-promoting residues, such as arginine, are less frequent than expected by chance (calculated from random nucleotides), while others, including lysine, are more frequent, see Fig 10 and S1 Fig. Therefore, it is unlikely that a purifying selection is the only driving force for the observed shifts in amino acid preferences between eukaryotes and prokaryotes.

In bacteria, one reason to reduce the frequency of an amino acid is the energetic cost to produce it [66]. Serine is among the least costly amino acids to make both aerobically and anaerobically [66, 67], S7 Table. Proline is cheaper than most amino acids to make, while isoleucine is among the most expensive ones. Therefore, the cost of producing amino acids would predict that serine and proline frequencies decreased in the species with higher selective pressure, i.e. the prokaryotes and isoleucine increased, opposite to what is observed. It has also been reported that high serine levels are toxic [68, 69], possibly contributing to reduced serine levels are reduced in prokaryotes.

Anyhow, none of the explanations discussed above can fully explain the shift in frequency for all three amino acids. Further, if there just was a selective pressure to decrease the amount of disorder, it is not clear why only the frequencies of three amino acids should be affected. Therefore, it is unlikely that the reduced selective pressure in eukaryotes can explain the shifts in amino acid frequencies.

### Conclusion

Here, we confirm earlier observations that eukaryotic proteins are more disordered than prokaryotic proteins. We show that more extended and more disordered linkers cause a systematic increase in intrinsic disorder in eukaryotic proteins. Further, we show that the increased disorder in the linkers originates from a systematic shift in the frequency of only three amino acids, serine, proline, and isoleucine. Serine and proline are more frequent in eukaryotic proteins than in prokaryotic proteins, while isoleucine is less frequent. For serine, the difference holds for all phyla, protein families, structural regions of proteins and type of protein but is most pronounced in disordered and linker regions. The proline and isoleucine differences are also observed in most classes of proteins and regions but are affected by differences in GC levels of the genomes. Anyhow, it is safe to assume that the differences in amino acid frequencies occurred soon after the three kingdoms split and have been maintained during the last billion years.

It is not clear if the increases of serine and proline and decrease in isoleucine cause the increased disorder in eukaryotic proteins, or are a consequence of it. It is tempting to speculate that the increase in serine is related to its importance as a target for regulatory kinases, but direct evidence for this is lacking. Further, the increased need for regulation in eukaryotes does not explain the shift in proline and isoleucine frequencies. Anyhow, the observation that not all disorder-promoting amino acids are increased in eukaryotic linkers makes it clear that earlier explanations of the increased disorder in eukaryotic proteins are too simplified. Further, why just isoleucine, serine and proline frequencies differ between eukaryotes and prokaryotes remains an open question that requires further analysis.

## Supporting information

S1 Table A two way ANOVA F-test for contribution of different amino acids to the difference between eukaryotic and bacterial proteomes when including the GC genomic content. Here, all genomes before filtering on GC are included.
(PDF)

S2 Table Summary of average features for different set of proteins and protein regions in Eukaryota. (PDF)

S3 Table Summary of average features for different set of proteins and protein regions in Bacteria. (PDF)

S4 Table Summary of average features for different set of proteins and protein regions in Archaea. (PDF)

S5 Table List of the GO terms that where the frequency differs with more than 1.5% between eukaryotes and bacteria for isoleucine, proline or serine. The GO terms are obtained from the Pfam domains and mapped to the GO-slim terms [50, 52]. NumSeq is the minimum number of sequences in a Pfam family; numPfam is that number of Pfam families with this GO term.
(PDF)

S6 Table Frequency of amino acids in different subcellular compartments. The sequences are taken from all Swissprot proteins with subcellular annotations. Each compartment is divided into a membrane and a non-membrane part as this is a major influence on amino acid frequencies. The amino acids are sorted by their one letter code,
(PDF)

S7 Table Anaerobic and aerobic costs to produce an amino acid. Data in first column is from Akashi et al [66] and in column two and three from Raiford et al [67]. The unit is the number of *PO*_4_ molecules to produce one amino acid. The amino acids are sorted according to the cost in the first column.
(PDF)

S1 Fig Frequency of all amino acids vs. GC of the genomes. The amino acids are sorted after the GC content of their codons. The number next to each figure represents the fraction of GC among the codons. Archaeal genomes are red, bacteria dark green, and eukaryotes are blue. The straight lines represent linear fits for each kingdom independently. Here, the data for genomes with GC higher than 60% and lower than 20% are also included for clarity.
(PDF)

S2 Fig Distribution of GC in genomes from different kingdoms. In (a) data for all genome are shown and in (b) only the genomes that remained after filtering for GC between 20% and 60%. When all genomes are present the average GC content of eukaryotes is 43.8%, 51.0% for bacteria and 47.2% for archaea. After filtering the average GC contents similar in all three kingdoms (43.2 to 44.0%) as are the standard deviations (8.0 to 8.4%). By filtering 2.6% of the eukaryotic genomes are excluded (25 out of 1001), 20% of the archaeal (75 out of 383) and 30% of the bacterial ones (2219 out of 7124).
(PDF)

S3 Fig Difference in disorder propensity contributed by differences in amino acid frequency in the linkers in the three kingdoms. The differences in propensities are calculated by multiplying the TOP-IDP propensity score with the difference in frequency between eukaryotes and one of the prokaryotes. Error bars represent the standard error for each amino acid. The amino acids are sorted according to their disorder propensity in the TOP-IDP scale.
(PDF)

S4 Fig Frequency of amino acids in linker regions grouped by phylum. Bacterial groups are red, eukaryotic dark green and archaeal blue. The amino acids are sorted by their one letter code.
(PDF)

S5 Fig Distribution of genomic GC content for different phylogenetic groups. Red is bacteria, blue archaea and dark green eukaryota. Only the genomes that remained after filtering are included here.
(PDF)

S6 Fig Frequency of amino acids in different secondary structures in bacterial and eukaryotic proteins. (PDF)
