# Supplementary material for: Why do eukaryotic proteins contain more intrinsically disordered regions?
Source: PLoS Comput Biol. 2019 Jul 22;15(7):e1007186. doi: 10.1371/journal.pcbi.1007186 (PMC6675126; doi:10.1371/journal.pcbi.1007186)
Supplement: S1 Table — Here, all genomes before filtering on GC are included. (PDF) [file pcbi.1007186.s001.pdf]

Supporting Information Legends

AA	F
Ser	3058
Pro	2488
Ile	2173
Cys	1169
His	749
Arg	510
Tyr	489
Met	450
Lys	410
Phe	364
Leu	293
Val	238
Gln	181
Gly	157
Ala	143
Asn	81
Thr	60
Gly	42
Asp	38
Trp	23

Table S1. ANOVA F-test for contribution of different amino acids to the difference between eukaryotic and bacterial proteomes when including the GC genomic content. Here, all genomes before filtering on GC are included.
