# Supplementary material for: Why do eukaryotic proteins contain more intrinsically disordered regions?
Source: PLoS Comput Biol. 2019 Jul 22;15(7):e1007186. doi: 10.1371/journal.pcbi.1007186 (PMC6675126; doi:10.1371/journal.pcbi.1007186)
Supplement: S2 Table — (PDF) [file pcbi.1007186.s002.pdf]

Eukaryota	All proteins	Shared Proteins	Specific Proteins	No Domain proteins	Shared domains	Specific domains	Linker regions	N-terminal Linker regions	Central Linker regions	C-terminal Linker regions
IUpred long(AA)	0.324 ± 0.002	0.246 ± 0.002	0.386 ± 0.003	0.421 ± 0.003	0.116 ± 0.001	0.171 ± 0.002	0.381 ± 0.003	0.399 ± 0.003	0.341 ± 0.003	0.387 ± 0.003
IUpred short(AA)	0.289 ± 0.002	0.223 ± 0.001	0.338 ± 0.002	0.375 ± 0.002	0.093 ± 0.001	0.131 ± 0.001	0.363 ± 0.002	0.393 ± 0.003	0.301 ± 0.002	0.371 ± 0.003
<SEG>	0.087 ± 0.001	0.067 ± 0.001	0.101 ± 0.001	0.112 ± 0.001	0.035 ± 0.0	0.04 ± 0.0	0.099 ± 0.001	0.106 ± 0.002	0.086 ± 0.001	0.101 ± 0.001
<TOP-IDP>	0.096 ± 0.001	0.085 ± 0.0	0.106 ± 0.001	0.112 ± 0.001	0.056 ± 0.0	0.073 ± 0.0	0.115 ± 0.001	0.114 ± 0.001	0.118 ± 0.001	0.115 ± 0.001
<Hydrophobicity> (Hessa)	1.08 ± 0.001	1.05 ± 0.001	1.113 ± 0.001	1.114 ± 0.001	0.974 ± 0.001	1.04 ± 0.001	1.125 ± 0.002	1.111 ± 0.002	1.133 ± 0.002	1.134 ± 0.001
Length (AA)	448.528 ± 3.297	532.192 ± 3.24	496.778 ± 3.003	333.891 ± 3.169	233.695 ± 0.008	49.004 ± 0.002	258.287 ± 0.009	98.304 ± 0.003	68.534 ± 0.003	91.449 ± 0.003
Number of disorder residues (long)	145.234 ± 1.008	130.704 ± 0.982	191.681 ± 1.358	140.731 ± 0.98	27.118 ± 0.266	8.401 ± 0.081	98.484 ± 0.808	39.218 ± 0.341	23.399 ± 0.203	35.388 ± 0.294
Number of disorder residue (short)	129.629 ± 0.795	118.646 ± 0.757	167.755 ± 1.115	125.236 ± 0.788	21.75 ± 0.175	6.431 ± 0.056	93.881 ± 0.642	38.624 ± 0.278	20.649 ± 0.169	33.97 ± 0.229
Low complexity residues	38.977 ± 0.527	35.572 ± 0.448	49.975 ± 0.522	37.313 ± 0.482	8.079 ± 0.099	1.978 ± 0.023	25.63 ± 0.356	10.378 ± 0.156	5.887 ± 0.082	9.223 ± 0.124
TRP	0.012 ± 0.0	0.013 ± 0.0	0.011 ± 0.0	0.012 ± 0.0	0.015 ± 0.0	0.013 ± 0.0	0.011 ± 0.0	0.011 ± 0.0	0.011 ± 0.0	0.013 ± 0.0
PHE	0.039 ± 0.0	0.041 ± 0.0	0.038 ± 0.0	0.036 ± 0.0	0.046 ± 0.0	0.043 ± 0.0	0.036 ± 0.0	0.036 ± 0.0	0.035 ± 0.0	0.036 ± 0.0
TYR	0.03 ± 0.0	0.031 ± 0.0	0.029 ± 0.0	0.027 ± 0.0	0.034 ± 0.0	0.033 ± 0.0	0.027 ± 0.0	0.027 ± 0.0	0.027 ± 0.0	0.028 ± 0.0
ILE	0.053 ± 0.0	0.056 ± 0.0	0.051 ± 0.0	0.05 ± 0.0	0.063 ± 0.0	0.058 ± 0.0	0.049 ± 0.0	0.048 ± 0.0	0.05 ± 0.0	0.049 ± 0.0
MET	0.022 ± 0.0	0.022 ± 0.0	0.022 ± 0.0	0.022 ± 0.0	0.023 ± 0.0	0.021 ± 0.0	0.022 ± 0.0	0.026 ± 0.0	0.02 ± 0.0	0.021 ± 0.0
LEU	0.092 ± 0.0	0.093 ± 0.0	0.093 ± 0.0	0.089 ± 0.0	0.098 ± 0.0	0.098 ± 0.0	0.088 ± 0.0	0.09 ± 0.0	0.088 ± 0.0	0.087 ± 0.0
VAL	0.061 ± 0.0	0.065 ± 0.0	0.057 ± 0.0	0.057 ± 0.0	0.071 ± 0.0	0.065 ± 0.0	0.058 ± 0.0	0.057 ± 0.0	0.06 ± 0.0	0.059 ± 0.0
ASN	0.045 ± 0.001	0.044 ± 0.0	0.045 ± 0.001	0.045 ± 0.001	0.042 ± 0.0	0.044 ± 0.0	0.045 ± 0.001	0.044 ± 0.001	0.046 ± 0.001	0.044 ± 0.001
CYS	0.017 ± 0.0	0.016 ± 0.0	0.016 ± 0.0	0.017 ± 0.0	0.018 ± 0.0	0.022 ± 0.0	0.015 ± 0.0	0.015 ± 0.0	0.015 ± 0.0	0.015 ± 0.0
THR	0.057 ± 0.0	0.056 ± 0.0	0.056 ± 0.0	0.059 ± 0.0	0.055 ± 0.0	0.054 ± 0.0	0.057 ± 0.0	0.058 ± 0.0	0.058 ± 0.0	0.055 ± 0.0
ALA	0.074 ± 0.001	0.075 ± 0.001	0.074 ± 0.001	0.074 ± 0.001	0.076 ± 0.001	0.074 ± 0.001	0.075 ± 0.001	0.076 ± 0.001	0.074 ± 0.001	0.074 ± 0.001
GLY	0.061 ± 0.0	0.067 ± 0.0	0.056 ± 0.0	0.058 ± 0.0	0.073 ± 0.0	0.061 ± 0.0	0.06 ± 0.0	0.056 ± 0.0	0.062 ± 0.0	0.063 ± 0.0
ARG	0.056 ± 0.0	0.054 ± 0.0	0.058 ± 0.0	0.06 ± 0.0	0.05 ± 0.0	0.055 ± 0.0	0.058 ± 0.0	0.059 ± 0.0	0.056 ± 0.0	0.059 ± 0.0
ASP	0.055 ± 0.0	0.055 ± 0.0	0.056 ± 0.0	0.054 ± 0.0	0.054 ± 0.0	0.055 ± 0.0	0.056 ± 0.0	0.055 ± 0.0	0.059 ± 0.0	0.057 ± 0.0
HIS	0.024 ± 0.0	0.024 ± 0.0	0.024 ± 0.0	0.026 ± 0.0	0.025 ± 0.0	0.026 ± 0.0	0.024 ± 0.0	0.025 ± 0.0	0.023 ± 0.0	0.024 ± 0.0
GLN	0.041 ± 0.0	0.038 ± 0.0	0.045 ± 0.0	0.044 ± 0.0	0.034 ± 0.0	0.039 ± 0.0	0.042 ± 0.0	0.042 ± 0.0	0.042 ± 0.0	0.043 ± 0.0
SER	0.084 ± 0.0	0.078 ± 0.0	0.087 ± 0.0	0.091 ± 0.0	0.068 ± 0.0	0.071 ± 0.0	0.089 ± 0.0	0.094 ± 0.0	0.086 ± 0.0	0.086 ± 0.0
LYS	0.057 ± 0.0	0.057 ± 0.0	0.059 ± 0.0	0.057 ± 0.001	0.053 ± 0.0	0.06 ± 0.0	0.058 ± 0.001	0.056 ± 0.001	0.059 ± 0.001	0.06 ± 0.001
GLU	0.064 ± 0.0	0.062 ± 0.0	0.068 ± 0.0	0.065 ± 0.0	0.057 ± 0.0	0.065 ± 0.0	0.067 ± 0.0	0.062 ± 0.0	0.069 ± 0.0	0.07 ± 0.0
PRO	0.054 ± 0.0	0.051 ± 0.0	0.054 ± 0.0	0.058 ± 0.0	0.044 ± 0.0	0.042 ± 0.0	0.059 ± 0.0	0.062 ± 0.001	0.059 ± 0.0	0.056 ± 0.0
<Alpha propensity>	-0.01 ± 0.0	-0.011 ± 0.0	-0.008 ± 0.0	-0.012 ± 0.0	-0.01 ± 0.0	-0.004 ± 0.0	-0.012 ± 0.0	-0.013 ± 0.0	-0.013 ± 0.0	-0.011 ± 0.0
<Beta propensity>	-0.045 ± 0.0	-0.042 ± 0.0	-0.049 ± 0.0	-0.05 ± 0.0	-0.032 ± 0.0	-0.036 ± 0.0	-0.053 ± 0.0	-0.052 ± 0.0	-0.054 ± 0.0	-0.053 ± 0.0
<Coil propensity>	-0.011 ± 0.0	-0.012 ± 0.0	-0.012 ± 0.0	-0.009 ± 0.0	-0.014 ± 0.0	-0.016 ± 0.0	-0.009 ± 0.0	-0.008 ± 0.0	-0.009 ± 0.0	-0.01 ± 0.0
<Turn propensity>	-0.055 ± 0.0	-0.06 ± 0.0	-0.052 ± 0.0	-0.05 ± 0.0	-0.07 ± 0.0	-0.065 ± 0.0	-0.048 ± 0.0	-0.049 ± 0.0	-0.048 ± 0.0	-0.047 ± 0.0

Table S2. Summary of average features for different set of proteins and protein regions in Eukaryota.
