# Supplementary material for: Why do eukaryotic proteins contain more intrinsically disordered regions?
Source: PLoS Comput Biol. 2019 Jul 22;15(7):e1007186. doi: 10.1371/journal.pcbi.1007186 (PMC6675126; doi:10.1371/journal.pcbi.1007186)
Supplement: S3 Table — (PDF) [file pcbi.1007186.s003.pdf]

Bacteria	All proteins	Shared Proteins	Specific Proteins	No Domain proteins	Shared domains	Specific domains	Linker regions	N-terminal Linker regions	Central Linker regions	C-terminal Linker regions
IUpred long(AA)	0.123 ± 0.001	0.113 ± 0.001	0.14 ± 0.001	0.149 ± 0.001	0.094 ± 0.001	0.095 ± 0.001	0.165 ± 0.001	0.145 ± 0.001	0.182 ± 0.001	0.172 ± 0.001
IUpred short(AA)	0.124 ± 0.0	0.114 ± 0.0	0.138 ± 0.001	0.154 ± 0.001	0.076 ± 0.0	0.075 ± 0.0	0.202 ± 0.001	0.219 ± 0.001	0.141 ± 0.001	0.223 ± 0.001
<SEG>	0.047 ± 0.0	0.043 ± 0.0	0.057 ± 0.0	0.061 ± 0.0	0.036 ± 0.0	0.036 ± 0.0	0.057 ± 0.0	0.068 ± 0.0	0.051 ± 0.0	0.051 ± 0.0
<TOP-IDP>	0.059 ± 0.0	0.06 ± 0.0	0.054 ± 0.0	0.055 ± 0.0	0.055 ± 0.0	0.059 ± 0.0	0.071 ± 0.0	0.051 ± 0.0	0.091 ± 0.0	0.08 ± 0.0
<Hydrophobicity> (Hessa)	0.998 ± 0.0	0.997 ± 0.0	0.993 ± 0.001	1.004 ± 0.001	0.974 ± 0.001	1.002 ± 0.001	1.051 ± 0.001	0.986 ± 0.001	1.099 ± 0.001	1.088 ± 0.001
Length (AA)	305.169 ± 0.482	344.522 ± 0.481	262.697 ± 0.7	218.984 ± 0.574	226.903 ± 0.001	26.798 ± 0.0	112.426 ± 0.0	43.838 ± 0.0	26.686 ± 0.0	41.902 ± 0.0
Number of disorder residues (long)	37.411 ± 0.166	38.88 ± 0.174	36.698 ± 0.229	32.62 ± 0.161	21.301 ± 0.124	2.558 ± 0.02	18.528 ± 0.117	6.374 ± 0.046	4.864 ± 0.032	7.224 ± 0.045
Number of disorder residue (short)	37.813 ± 0.123	39.287 ± 0.125	36.291 ± 0.161	33.827 ± 0.128	17.328 ± 0.082	2.021 ± 0.013	22.748 ± 0.097	9.609 ± 0.04	3.771 ± 0.024	9.325 ± 0.039
Low complexity residues	14.445 ± 0.043	14.785 ± 0.043	15.073 ± 0.059	13.276 ± 0.051	8.246 ± 0.032	0.973 ± 0.006	6.458 ± 0.03	2.965 ± 0.014	1.359 ± 0.008	2.12 ± 0.012
TRP	0.011 ± 0.0	0.011 ± 0.0	0.012 ± 0.0	0.013 ± 0.0	0.01 ± 0.0	0.01 ± 0.0	0.011 ± 0.0	0.011 ± 0.0	0.009 ± 0.0	0.012 ± 0.0
PHE	0.044 ± 0.0	0.044 ± 0.0	0.047 ± 0.0	0.048 ± 0.0	0.043 ± 0.0	0.042 ± 0.0	0.042 ± 0.0	0.046 ± 0.0	0.038 ± 0.0	0.041 ± 0.0
TYR	0.034 ± 0.0	0.033 ± 0.0	0.036 ± 0.0	0.038 ± 0.0	0.034 ± 0.0	0.035 ± 0.0	0.034 ± 0.0	0.033 ± 0.0	0.032 ± 0.0	0.035 ± 0.0
ILE	0.073 ± 0.0	0.074 ± 0.0	0.072 ± 0.0	0.071 ± 0.0	0.075 ± 0.0	0.072 ± 0.0	0.069 ± 0.0	0.071 ± 0.0	0.067 ± 0.0	0.067 ± 0.0
MET	0.024 ± 0.0	0.025 ± 0.0	0.024 ± 0.0	0.024 ± 0.0	0.024 ± 0.0	0.021 ± 0.0	0.029 ± 0.0	0.042 ± 0.0	0.02 ± 0.0	0.021 ± 0.0
LEU	0.099 ± 0.0	0.099 ± 0.0	0.1 ± 0.0	0.098 ± 0.0	0.098 ± 0.0	0.099 ± 0.0	0.097 ± 0.0	0.102 ± 0.0	0.093 ± 0.0	0.096 ± 0.0
VAL	0.068 ± 0.0	0.069 ± 0.0	0.067 ± 0.0	0.063 ± 0.0	0.072 ± 0.0	0.07 ± 0.0	0.062 ± 0.0	0.061 ± 0.0	0.065 ± 0.0	0.062 ± 0.0
ASN	0.045 ± 0.0	0.043 ± 0.0	0.048 ± 0.0	0.049 ± 0.0	0.042 ± 0.0	0.046 ± 0.0	0.048 ± 0.0	0.048 ± 0.0	0.048 ± 0.0	0.049 ± 0.0
CYS	0.01 ± 0.0	0.01 ± 0.0	0.008 ± 0.0	0.011 ± 0.0	0.01 ± 0.0	0.01 ± 0.0	0.008 ± 0.0	0.009 ± 0.0	0.007 ± 0.0	0.009 ± 0.0
THR	0.054 ± 0.0	0.053 ± 0.0	0.056 ± 0.0	0.055 ± 0.0	0.053 ± 0.0	0.054 ± 0.0	0.055 ± 0.0	0.056 ± 0.0	0.056 ± 0.0	0.052 ± 0.0
ALA	0.079 ± 0.0	0.08 ± 0.0	0.077 ± 0.0	0.075 ± 0.0	0.084 ± 0.0	0.082 ± 0.0	0.075 ± 0.0	0.073 ± 0.0	0.079 ± 0.0	0.075 ± 0.0
GLY	0.07 ± 0.0	0.071 ± 0.0	0.066 ± 0.0	0.064 ± 0.0	0.076 ± 0.0	0.072 ± 0.0	0.06 ± 0.0	0.056 ± 0.0	0.064 ± 0.0	0.062 ± 0.0
ARG	0.048 ± 0.0	0.048 ± 0.0	0.046 ± 0.0	0.046 ± 0.0	0.047 ± 0.0	0.046 ± 0.0	0.049 ± 0.0	0.046 ± 0.0	0.049 ± 0.0	0.051 ± 0.0
ASP	0.053 ± 0.0	0.053 ± 0.0	0.052 ± 0.0	0.053 ± 0.0	0.054 ± 0.0	0.057 ± 0.0	0.054 ± 0.0	0.049 ± 0.0	0.061 ± 0.0	0.056 ± 0.0
HIS	0.019 ± 0.0	0.02 ± 0.0	0.017 ± 0.0	0.018 ± 0.0	0.021 ± 0.0	0.019 ± 0.0	0.018 ± 0.0	0.017 ± 0.0	0.019 ± 0.0	0.019 ± 0.0
GLN	0.036 ± 0.0	0.036 ± 0.0	0.039 ± 0.0	0.038 ± 0.0	0.035 ± 0.0	0.038 ± 0.0	0.04 ± 0.0	0.039 ± 0.0	0.042 ± 0.0	0.041 ± 0.0
SER	0.062 ± 0.0	0.061 ± 0.0	0.066 ± 0.0	0.067 ± 0.0	0.059 ± 0.0	0.061 ± 0.0	0.065 ± 0.0	0.068 ± 0.0	0.064 ± 0.0	0.063 ± 0.0
LYS	0.066 ± 0.0	0.066 ± 0.0	0.065 ± 0.0	0.067 ± 0.0	0.06 ± 0.0	0.063 ± 0.0	0.072 ± 0.0	0.071 ± 0.0	0.068 ± 0.0	0.076 ± 0.0
GLU	0.065 ± 0.0	0.066 ± 0.0	0.064 ± 0.0	0.064 ± 0.0	0.064 ± 0.0	0.066 ± 0.0	0.07 ± 0.0	0.062 ± 0.0	0.077 ± 0.0	0.074 ± 0.0
PRO	0.039 ± 0.0	0.04 ± 0.0	0.038 ± 0.0	0.039 ± 0.0	0.039 ± 0.0	0.036 ± 0.0	0.04 ± 0.0	0.039 ± 0.0	0.043 ± 0.0	0.039 ± 0.0
<Alpha propensity>	-0.004 ± 0.0	-0.004 ± 0.0	-0.004 ± 0.0	-0.005 ± 0.0	-0.005 ± 0.0	-0.004 ± 0.0	-0.001 ± 0.0	0.0 ± 0.0	-0.003 ± 0.0	-0.001 ± 0.0
<Beta propensity>	-0.033 ± 0.0	-0.033 ± 0.0	-0.031 ± 0.0	-0.032 ± 0.0	-0.032 ± 0.0	-0.033 ± 0.0	-0.037 ± 0.0	-0.029 ± 0.0	-0.045 ± 0.0	-0.041 ± 0.0
<Coil propensity>	-0.018 ± 0.0	-0.018 ± 0.0	-0.017 ± 0.0	-0.017 ± 0.0	-0.017 ± 0.0	-0.017 ± 0.0	-0.018 ± 0.0	-0.019 ± 0.0	-0.016 ± 0.0	-0.018 ± 0.0
<Turn propensity>	-0.074 ± 0.0	-0.075 ± 0.0	-0.075 ± 0.0	-0.072 ± 0.0	-0.076 ± 0.0	-0.073 ± 0.0	-0.07 ± 0.0	-0.079 ± 0.0	-0.063 ± 0.0	-0.065 ± 0.0

Table S3. Summary of average features for different set of proteins and protein regions in Bacteria.
