# Supplementary material for: Why do eukaryotic proteins contain more intrinsically disordered regions?
Source: PLoS Comput Biol. 2019 Jul 22;15(7):e1007186. doi: 10.1371/journal.pcbi.1007186 (PMC6675126; doi:10.1371/journal.pcbi.1007186)
Supplement: S4 Table — (PDF) [file pcbi.1007186.s004.pdf]

Archaea	All proteins	Shared Proteins	Specific Proteins	No Domain proteins	Shared domains	Specific domains	Linker regions	N-terminal Linker regions	Central Linker regions	C-terminal Linker regions
IUpred long(AA)	0.122 ± 0.005	0.116 ± 0.005	0.127 ± 0.006	0.137 ± 0.006	0.076 ± 0.005	0.069 ± 0.005	0.116 ± 0.006	0.104 ± 0.006	0.119 ± 0.007	0.125 ± 0.007
IUpred short(AA)	0.127 ± 0.004	0.118 ± 0.003	0.135 ± 0.004	0.148 ± 0.004	0.064 ± 0.003	0.057 ± 0.003	0.171 ± 0.005	0.197 ± 0.004	0.094 ± 0.005	0.18 ± 0.005
<SEG>	0.052 ± 0.001	0.047 ± 0.001	0.054 ± 0.001	0.07 ± 0.001	0.042 ± 0.001	0.039 ± 0.001	0.057 ± 0.001	0.063 ± 0.001	0.048 ± 0.001	0.055 ± 0.001
<TOP-IDP>	0.067 ± 0.001	0.069 ± 0.001	0.07 ± 0.001	0.058 ± 0.001	0.058 ± 0.001	0.067 ± 0.001	0.07 ± 0.001	0.055 ± 0.001	0.09 ± 0.001	0.076 ± 0.001
<Hydrophobicity> (Hessa)	1.014 ± 0.003	1.016 ± 0.003	1.028 ± 0.004	1.005 ± 0.004	0.966 ± 0.003	1.015 ± 0.003	1.041 ± 0.003	0.994 ± 0.004	1.096 ± 0.005	1.062 ± 0.004
Length (AA)	270.13 ± 1.915	309.482 ± 1.776	251.14 ± 3.406	198.698 ± 1.985	218.588 ± 0.045	18.757 ± 0.005	106.084 ± 0.026	40.426 ± 0.01	19.699 ± 0.006	45.959 ± 0.01
Number of disorder residues (long)	32.984 ± 1.33	35.925 ± 1.45	31.97 ± 1.44	27.163 ± 1.093	16.671 ± 0.995	1.303 ± 0.099	12.291 ± 0.689	4.224 ± 0.251	2.34 ± 0.134	5.726 ± 0.316
Number of disorder residue (short)	34.203 ± 0.951	36.62 ± 0.995	33.999 ± 1.041	29.416 ± 0.835	13.986 ± 0.629	1.07 ± 0.062	18.119 ± 0.483	7.956 ± 0.18	1.849 ± 0.095	8.287 ± 0.234
Low complexity residues	14.175 ± 0.184	14.419 ± 0.185	13.517 ± 0.305	13.834 ± 0.203	9.083 ± 0.158	0.734 ± 0.022	6.015 ± 0.127	2.546 ± 0.059	0.936 ± 0.025	2.513 ± 0.056
TRP	0.01 ± 0.0	0.01 ± 0.0	0.01 ± 0.0	0.012 ± 0.0	0.01 ± 0.0	0.008 ± 0.0	0.011 ± 0.0	0.011 ± 0.0	0.01 ± 0.0	0.011 ± 0.0
PHE	0.041 ± 0.0	0.04 ± 0.0	0.042 ± 0.0	0.044 ± 0.0	0.04 ± 0.0	0.038 ± 0.0	0.039 ± 0.0	0.041 ± 0.0	0.036 ± 0.0	0.038 ± 0.001
TYR	0.036 ± 0.0	0.035 ± 0.0	0.036 ± 0.0	0.04 ± 0.0	0.036 ± 0.0	0.036 ± 0.0	0.038 ± 0.001	0.038 ± 0.001	0.036 ± 0.001	0.039 ± 0.001
ILE	0.079 ± 0.001	0.079 ± 0.001	0.078 ± 0.001	0.079 ± 0.001	0.081 ± 0.001	0.079 ± 0.001	0.078 ± 0.001	0.08 ± 0.001	0.076 ± 0.001	0.077 ± 0.001
MET	0.025 ± 0.0	0.025 ± 0.0	0.024 ± 0.0	0.024 ± 0.0	0.023 ± 0.0	0.02 ± 0.0	0.03 ± 0.0	0.043 ± 0.0	0.02 ± 0.0	0.021 ± 0.0
LEU	0.094 ± 0.0	0.094 ± 0.0	0.096 ± 0.001	0.097 ± 0.001	0.095 ± 0.001	0.095 ± 0.001	0.095 ± 0.001	0.096 ± 0.001	0.091 ± 0.001	0.096 ± 0.001
VAL	0.073 ± 0.001	0.074 ± 0.001	0.073 ± 0.001	0.069 ± 0.001	0.079 ± 0.001	0.078 ± 0.001	0.071 ± 0.001	0.071 ± 0.001	0.072 ± 0.001	0.07 ± 0.001
ASN	0.043 ± 0.001	0.041 ± 0.001	0.042 ± 0.001	0.047 ± 0.001	0.039 ± 0.001	0.045 ± 0.001	0.044 ± 0.001	0.044 ± 0.001	0.044 ± 0.001	0.043 ± 0.001
CYS	0.011 ± 0.0	0.01 ± 0.0	0.01 ± 0.0	0.011 ± 0.0	0.011 ± 0.0	0.012 ± 0.0	0.009 ± 0.0	0.01 ± 0.0	0.008 ± 0.0	0.009 ± 0.0
THR	0.051 ± 0.0	0.05 ± 0.0	0.05 ± 0.001	0.053 ± 0.0	0.05 ± 0.0	0.048 ± 0.001	0.049 ± 0.001	0.049 ± 0.001	0.05 ± 0.001	0.048 ± 0.001
ALA	0.069 ± 0.001	0.071 ± 0.001	0.067 ± 0.001	0.063 ± 0.001	0.078 ± 0.001	0.075 ± 0.001	0.064 ± 0.001	0.061 ± 0.001	0.066 ± 0.001	0.065 ± 0.001
GLY	0.072 ± 0.0	0.074 ± 0.0	0.068 ± 0.001	0.066 ± 0.0	0.079 ± 0.0	0.077 ± 0.001	0.065 ± 0.001	0.063 ± 0.001	0.068 ± 0.001	0.067 ± 0.001
ARG	0.051 ± 0.001	0.052 ± 0.001	0.053 ± 0.001	0.049 ± 0.001	0.051 ± 0.001	0.054 ± 0.001	0.056 ± 0.001	0.053 ± 0.001	0.058 ± 0.001	0.058 ± 0.001
ASP	0.054 ± 0.0	0.055 ± 0.0	0.054 ± 0.001	0.053 ± 0.001	0.054 ± 0.001	0.058 ± 0.001	0.054 ± 0.001	0.052 ± 0.001	0.059 ± 0.001	0.054 ± 0.001
HIS	0.017 ± 0.0	0.017 ± 0.0	0.016 ± 0.0	0.015 ± 0.0	0.018 ± 0.0	0.017 ± 0.0	0.016 ± 0.0	0.015 ± 0.0	0.016 ± 0.0	0.016 ± 0.0
GLN	0.024 ± 0.0	0.024 ± 0.0	0.025 ± 0.0	0.026 ± 0.0	0.022 ± 0.0	0.022 ± 0.0	0.024 ± 0.0	0.023 ± 0.0	0.025 ± 0.001	0.025 ± 0.0
SER	0.064 ± 0.0	0.062 ± 0.0	0.064 ± 0.001	0.07 ± 0.0	0.059 ± 0.001	0.059 ± 0.001	0.064 ± 0.001	0.065 ± 0.001	0.061 ± 0.001	0.063 ± 0.001
LYS	0.07 ± 0.001	0.071 ± 0.001	0.072 ± 0.001	0.068 ± 0.001	0.062 ± 0.001	0.067 ± 0.002	0.073 ± 0.002	0.07 ± 0.002	0.073 ± 0.002	0.076 ± 0.002
GLU	0.076 ± 0.001	0.076 ± 0.001	0.079 ± 0.001	0.074 ± 0.001	0.07 ± 0.001	0.075 ± 0.001	0.079 ± 0.001	0.073 ± 0.001	0.085 ± 0.001	0.082 ± 0.001
PRO	0.04 ± 0.0	0.041 ± 0.0	0.041 ± 0.0	0.039 ± 0.0	0.042 ± 0.0	0.037 ± 0.0	0.043 ± 0.001	0.042 ± 0.001	0.047 ± 0.001	0.042 ± 0.001
<Alpha propensity>	-0.005 ± 0.0	-0.005 ± 0.0	-0.004 ± 0.0	-0.006 ± 0.0	-0.007 ± 0.0	-0.005 ± 0.0	-0.004 ± 0.0	-0.003 ± 0.0	-0.005 ± 0.001	-0.003 ± 0.0
<Beta propensity>	-0.032 ± 0.0	-0.033 ± 0.0	-0.032 ± 0.001	-0.029 ± 0.001	-0.03 ± 0.0	-0.033 ± 0.0	-0.033 ± 0.001	-0.028 ± 0.001	-0.041 ± 0.001	-0.035 ± 0.001
<Coil propensity>	-0.018 ± 0.0	-0.018 ± 0.0	-0.019 ± 0.0	-0.018 ± 0.0	-0.018 ± 0.0	-0.018 ± 0.0	-0.019 ± 0.0	-0.019 ± 0.0	-0.017 ± 0.0	-0.019 ± 0.0
<Turn propensity>	-0.076 ± 0.0	-0.076 ± 0.0	-0.076 ± 0.001	-0.076 ± 0.001	-0.081 ± 0.0	-0.075 ± 0.0	-0.076 ± 0.001	-0.084 ± 0.001	-0.069 ± 0.001	-0.073 ± 0.001

Table S4. Summary of average features for different set of proteins and protein regions in Archaea.
