# Supplementary material for: Why do eukaryotic proteins contain more intrinsically disordered regions?
Source: PLoS Comput Biol. 2019 Jul 22;15(7):e1007186. doi: 10.1371/journal.pcbi.1007186 (PMC6675126; doi:10.1371/journal.pcbi.1007186)
Supplement: S5 Table — The GO terms are obtained from the Pfam domains and mapped to the GO-slim terms [50, 52]. NumSeq is the minimum number of sequences in a Pfam family; numPfam is that number of Pfam families with this GO term. (PDF) [file pcbi.1007186.s005.pdf]

NumSeq	GO	aa	bac_freq	euk_freq	numPfam	difference
951	GO:0003924	I	8.0%	6.0%	4.0	-2.0%
1071	GO:0004386	I	7.6%	5.7%	5.0	-2.0%
691	GO:0042592	I	7.1%	5.3%	4.0	-1.8%
771	GO:0006464	I	7.4%	5.8%	14.0	-1.7%
1436	GO:0051604	P	3.8%	2.2%	2.0	-1.6%
1539	GO:0005694	S	5.1%	6.7%	4.0	1.5%
959	GO:0003924	S	5.3%	7.1%	4.0	1.9%
339	GO:0008135	S	7.0%	4.3%	3.0	-2.7%
1439	GO:0051604	S	7.8%	6.3%	2.0	-1.5%

Table S5. List of the GO terms that where the frequency differs with more than 1.5% between eukaryotes and bacteria for isoleucine, proline or serine. The GO terms are obtained from the Pfam domains and mapped to the GO-slim terms [50, 52]. NumSeq is the minimum number of sequences in a Pfam family; numPfam is that number of Pfam families with this GO term.
