# Supplementary material for: Why do eukaryotic proteins contain more intrinsically disordered regions?
Source: PLoS Comput Biol. 2019 Jul 22;15(7):e1007186. doi: 10.1371/journal.pcbi.1007186 (PMC6675126; doi:10.1371/journal.pcbi.1007186)
Supplement: S6 Table — The sequences are taken from all Swissprot proteins with subcellular annotations. Each compartment is divided into a membrane and a non-membrane part as this is a major influence on amino acid frequencies. The amino acids are sorted by their one letter code, (PDF) [file pcbi.1007186.s006.pdf]

Compartment	Ala	Cys	Asp	Glu	Phe	Glu	His	Ile	Lys	Leu	Met	Asn	Pro	Gln	Arg	Ser	Thr	Val	Trp	Tyr
Endosome	6.9%	1.7%	5.4%	7.8%	3.7%	5.4%	2.3%	5.0%	6.0%	10.6%	2.3%	4.0%	5.0%	5.1%	5.4%	8.4%	5.0%	6.1%	1.1%	2.7%
ER	7.1%	1.4%	6.3%	7.8%	4.6%	6.4%	2.3%	4.9%	6.8%	9.9%	2.0%	3.9%	4.8%	3.8%	4.7%	6.9%	5.1%	6.6%	1.3%	3.3%
Mitochondrion	7.4%	1.5%	5.1%	6.4%	3.8%	6.5%	2.3%	5.5%	6.7%	9.8%	2.4%	4.3%	4.9%	3.9%	5.8%	7.4%	5.4%	6.6%	1.1%	2.9%
Golgi	6.0%	1.4%	5.5%	7.0%	4.1%	5.1%	2.2%	6.0%	6.4%	10.4%	2.2%	5.2%	4.6%	4.7%	4.6%	8.9%	5.3%	6.2%	1.0%	2.9%
Vacuole	6.7%	1.6%	5.5%	5.5%	4.9%	6.6%	2.1%	6.2%	5.4%	9.9%	2.1%	5.1%	4.6%	3.5%	4.1%	9.0%	5.5%	6.4%	1.5%	3.6%
Lysosome	6.6%	1.9%	4.9%	5.1%	4.4%	7.4%	2.7%	4.7%	4.7%	10.1%	2.3%	4.8%	5.6%	4.2%	4.5%	7.4%	5.6%	6.5%	2.3%	4.2%
Plastid	6.8%	1.4%	5.4%	6.6%	4.5%	6.5%	2.1%	5.9%	6.1%	9.6%	2.4%	4.3%	4.7%	3.3%	5.8%	8.7%	4.9%	6.4%	1.2%	3.0%
Cytoplasm	7.0%	1.6%	5.6%	7.7%	3.6%	6.1%	2.4%	5.1%	6.6%	9.5%	2.2%	4.3%	5.1%	4.5%	5.3%	7.8%	5.3%	6.2%	1.0%	2.8%
Peroxisome	7.6%	1.6%	5.3%	6.1%	4.2%	7.1%	2.7%	5.5%	5.9%	9.6%	2.3%	4.2%	5.5%	3.7%	5.4%	7.0%	5.2%	7.1%	1.2%	3.0%
Nucleus	6.7%	1.8%	5.5%	7.4%	3.4%	5.9%	2.7%	4.5%	6.9%	8.7%	2.2%	4.4%	5.9%	4.9%	5.8%	8.9%	5.3%	5.4%	0.9%	2.6%
Extracellular	7.1%	3.2%	5.3%	5.2%	3.9%	8.5%	2.2%	4.5%	5.2%	8.3%	1.9%	4.7%	6.0%	3.8%	4.7%	7.8%	6.1%	6.2%	1.5%	3.4%
Archaea	7.6%	0.9%	5.9%	8.6%	3.3%	7.4%	1.8%	7.4%	7.1%	8.5%	2.4%	3.6%	4.2%	2.2%	5.7%	5.1%	4.7%	7.9%	0.8%	3.3%
Bacteria	9.3%	1.0%	5.8%	7.0%	3.5%	7.5%	2.2%	6.1%	5.6%	9.3%	2.4%	3.7%	4.2%	3.8%	5.8%	5.3%	5.1%	7.2%	0.9%	2.7%
Membrane																				
Peroxisome	6.7%	1.3%	4.5%	6.1%	4.2%	5.9%	2.0%	5.6%	6.2%	11.4%	2.3%	4.5%	4.5%	4.2%	5.7%	8.5%	5.1%	6.3%	1.5%	3.4%
Plasma	7.1%	2.2%	4.7%	5.9%	4.5%	6.6%	2.2%	5.5%	4.9%	10.2%	2.3%	4.2%	5.3%	3.9%	5.0%	8.3%	5.8%	6.8%	1.4%	3.2%
Vacuole	7.0%	0.9%	5.5%	5.5%	4.8%	6.5%	2.3%	5.9%	4.7%	9.9%	1.9%	4.6%	4.8%	3.3%	5.0%	9.2%	5.9%	7.1%	1.7%	3.5%
Mitochondrion	7.2%	1.2%	3.7%	4.3%	5.6%	6.4%	2.5%	7.2%	4.6%	12.6%	3.3%	4.2%	5.1%	3.1%	4.3%	7.3%	6.1%	5.9%	2.0%	3.5%
ER	6.9%	1.5%	4.6%	5.7%	5.3%	6.0%	2.4%	5.7%	5.3%	11.0%	2.4%	4.0%	5.1%	3.7%	4.9%	7.7%	5.4%	6.8%	1.7%	3.5%
Nuclear	7.1%	1.4%	5.3%	7.3%	4.0%	5.2%	2.0%	5.0%	6.2%	10.4%	1.9%	4.5%	4.9%	4.8%	5.0%	9.6%	5.8%	6.0%	1.0%	2.4%
Endosome	7.1%	1.5%	5.1%	6.8%	3.9%	5.4%	2.1%	5.2%	5.8%	10.1%	2.2%	4.6%	5.9%	5.4%	4.9%	8.6%	5.1%	5.9%	1.1%	3.1%
Plastid	7.0%	1.0%	3.6%	5.0%	6.3%	7.5%	1.9%	8.2%	4.3%	11.3%	2.5%	4.3%	4.4%	3.3%	4.4%	7.3%	5.5%	6.2%	1.9%	3.6%
Golgi	6.5%	1.8%	5.4%	5.9%	5.0%	6.1%	2.6%	5.3%	5.5%	10.3%	2.3%	4.3%	5.0%	3.8%	5.5%	7.5%	5.2%	6.4%	1.8%	3.5%
Lysosome	6.9%	2.2%	4.4%	5.2%	5.0%	6.4%	2.3%	5.5%	4.0%	1.12%	2.3%	3.9%	5.3%	4.0%	4.9%	8.1%	5.7%	7.1%	1.9%	3.4%
Archaea	7.8%	0.8%	5.6%	7.9%	3.6%	7.5%	1.7%	7.8%	6.8%	9.0%	2.4%	3.6%	4.1%	2.1%	5.4%	5.3%	4.8%	7.9%	0.8%	3.4%
Bacteria	9.4%	0.9%	5.2%	6.2%	4.0%	7.6%	2.1%	6.5%	5.3%	10.0%	2.6%	3.6%	4.1%	3.6%	5.4%	5.4%	5.1%	7.4%	1.1%	2.7%

Table S6. Frequency of amino acids in different subcellular compartments. The sequences are taken from all Swissprot proteins with subcellular annotations. Each compartment is divided into a membrane and a non-membrane part as this is a major influence on amino acid frequencies. The amino acids are sorted by their one letter code,
