# Supplementary material for: Why do eukaryotic proteins contain more intrinsically disordered regions?
Source: PLoS Comput Biol. 2019 Jul 22;15(7):e1007186. doi: 10.1371/journal.pcbi.1007186 (PMC6675126; doi:10.1371/journal.pcbi.1007186)
Supplement: S7 Table — Data in first column is from Akashi et al [66] and in column two and three from Raiford et al [67]. The unit is the number of PO4 molecules to produce one amino acid. The amino acids are sorted according to the cost in the first column. (PDF) [file pcbi.1007186.s007.pdf]

AA	Aerobic [66]	Aerobic [67]	Anaerobic [67]
Gly	11.7	14.5	1.0
Ser	11.7	14.5	1.0
Ala	11.7	14.5	2.0
Asp	12.7	15.5	3.0
Asn	14.7	18.5	6.0
Glu	15.3	9.5	2.0
Gln	16.3	10.5	3.0
Thr	18.7	21.5	9.0
Pro	20.3	14.5	7.0
Val	23.3	29.0	4.0
Cys	24.7	26.5	13.0
Arg	27.3	20.5	13.0
Leu	27.3	37.0	4.0
Lys	30.3	36.0	12.0
Ile	32.3	38.0	14.0
Met	34.3	36.5	24.0
His	38.3	29.0	5.0
Tyr	50.0	59.0	8.0
Phe	52.0	61.0	10.0
Trp	74.3	75.5	14.0

Table S7. Anaerobic and aerobic costs to produce an amino acid. Data in first column is from Akashi et al [66] and in column two and three from Raiford et al [67]. The unit is the number of PO_4 molecules to produce one amino acid. The amino acids are sorted according to the cost in the first column.
