# Supplementary material for: Why do eukaryotic proteins contain more intrinsically disordered regions?
Source: PLoS Comput Biol. 2019 Jul 22;15(7):e1007186. doi: 10.1371/journal.pcbi.1007186 (PMC6675126; doi:10.1371/journal.pcbi.1007186)
Supplement: S1 Fig — The amino acids are sorted after the GC content of their codons. The number next to each figure represents the fraction of GC among the codons. Archaeal genomes are red, bacteria dark green, and eukaryotes are blue. The straight lines represent linear fits for each kingdom independently. Here, the data for genomes with GC higher than 60% and lower than 20% are also included for clarity. (PDF) [file pcbi.1007186.s008.pdf]

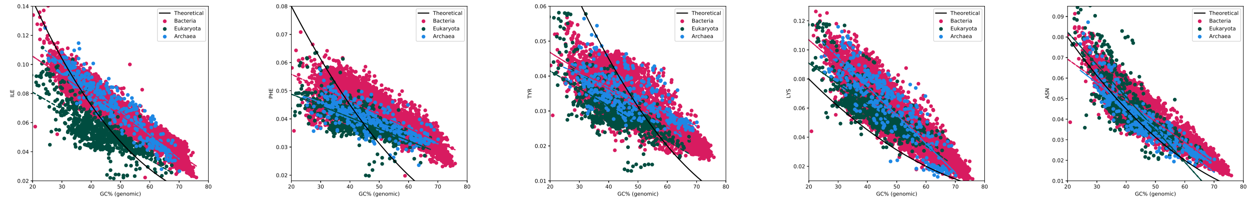

(a) 11% Ile **(b)** 17% Phe **(c)** 17% Tyr **(d)** 17% Lys **(e)** 17% Asn

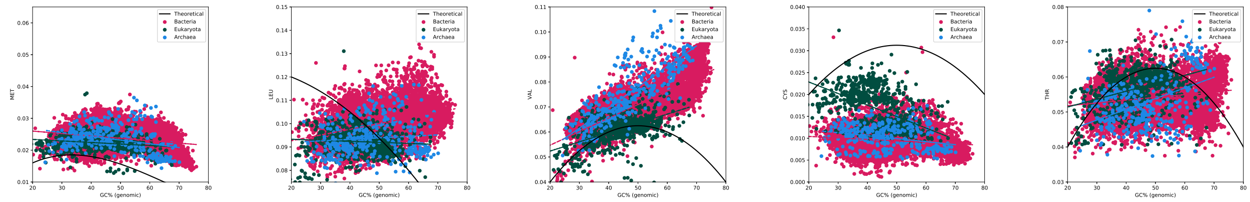

(f) 33% Met **(g)** 39% Leu **(h)** 50% Val **(i)** 50% Cys **(j)** 50% Thr

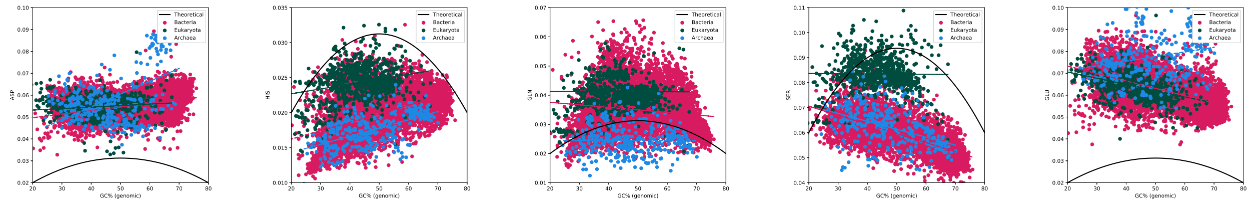

(k) 50% Asp **(l)** 50% His **(m)** 50% Gln **(n)** 50% Ser **(o)** 50% Glu

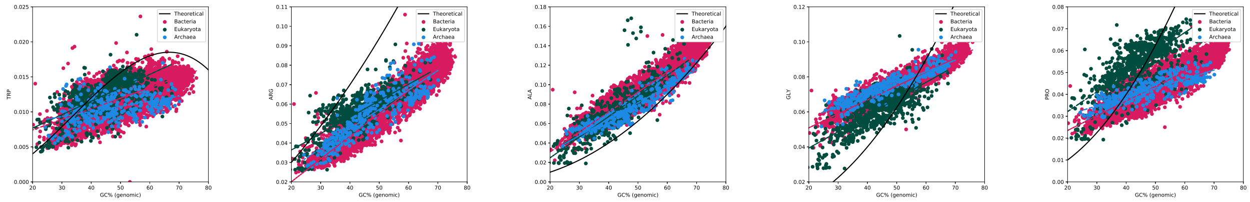

(p) 67% Trp **(q)** 72% Arg **(r)** 83% Ala **(s)** 83% Gly **(t)** 83% Pro

Figure S1. Frequency of all amino acids vs. GC of the genomes. The amino acids are sorted after the GC content of their codons. The number next to each figure represents the fraction of GC among the codons. Archaeal genomes are red, bacteria dark green, and eukaryotes are blue. The straight lines represent linear fits for each kingdom independently. Here, the data for genomes with GC higher than 60% and lower than 20% are also included for clarity.
