# Supplementary material for: Why do eukaryotic proteins contain more intrinsically disordered regions?
Source: PLoS Comput Biol. 2019 Jul 22;15(7):e1007186. doi: 10.1371/journal.pcbi.1007186 (PMC6675126; doi:10.1371/journal.pcbi.1007186)
Supplement: S2 Fig — In (a) data for all genome are shown and in (b) only the genomes that remained after filtering for GC between 20% and 60%. When all genomes are present the average GC content of eukaryotes is 43.8%, 51.0% for bacteria and 47.2% for archaea. After filtering the average GC contents similar in all three kingdoms (43.2 to 44.0%) as are the standard deviations (8.0 to 8.4%). By filtering 2.6% of the eukaryotic genomes are excluded (25 out of 1001), 20% of the archaeal (75 out of 383) and 30% of the bacterial ones (2219 out of 7124). (PDF) [file pcbi.1007186.s009.pdf]

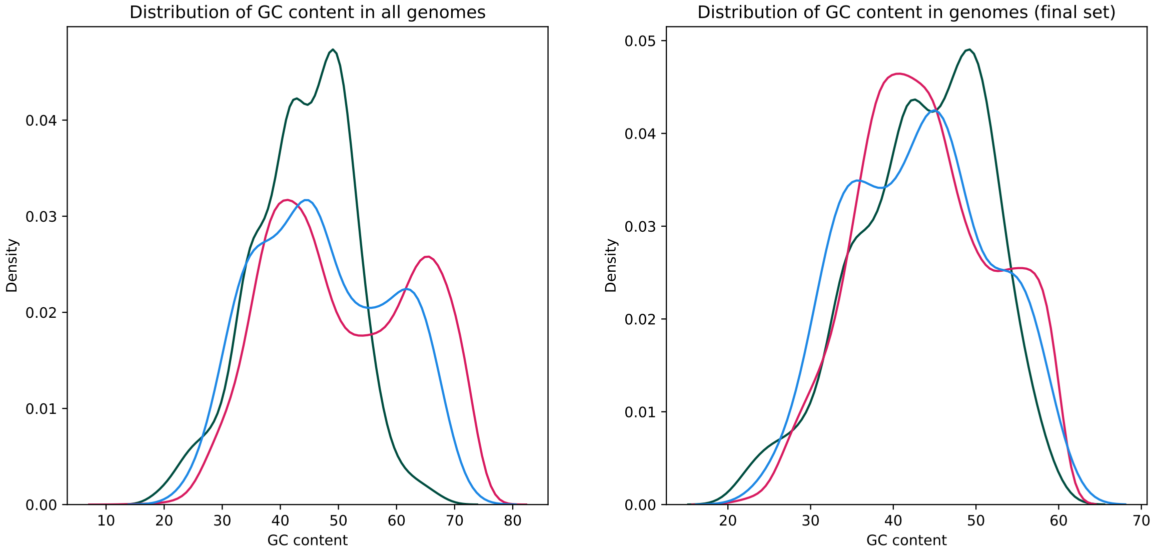

(a) **(b)**

Figure S2. Distribution of GC in genomes from different kingdoms. In (a) data for all genome are shown and in (b) only the genomes that remained after filtering for GC between 20% and 60%. When all genomes are present the average GC content of eukaryotes is 43.8%, 51.0% for bacteria and 47.2% for archaea. After filtering the average GC contents similar in all three kingdoms (43.2 to 44.0%) as are the standard deviations (8.0 to 8.4%). By filtering 2.6% of the eukaryotic genomes are excluded (25 out of 1001), 20% of the archaeal (75 out of 383) and 30% of the bacterial ones (2219 out of 7124).
