# Supplementary material for: Why do eukaryotic proteins contain more intrinsically disordered regions?
Source: PLoS Comput Biol. 2019 Jul 22;15(7):e1007186. doi: 10.1371/journal.pcbi.1007186 (PMC6675126; doi:10.1371/journal.pcbi.1007186)
Supplement: S3 Fig — The differences in propensities are calculated by multiplying the TOP-IDP propensity score with the difference in frequency between eukaryotes and one of the prokaryotes. Error bars represent the standard error for each amino acid. The amino acids are sorted according to their disorder propensity in the TOP-IDP scale. (PDF) [file pcbi.1007186.s010.pdf]

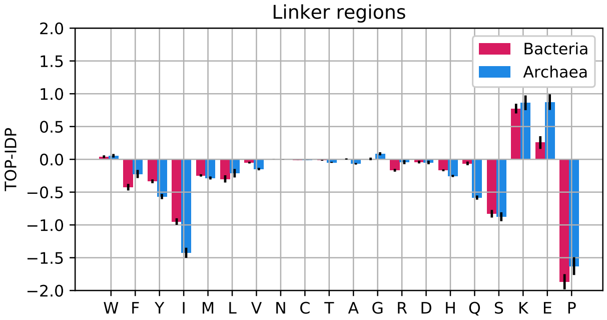

(a)

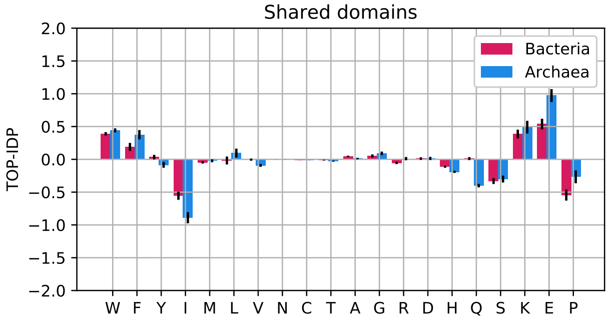

(b)

Figure S3. Difference in disorder propensity contributed by differences in amino acid frequency in the linkers in the three kingdoms. The differences in propensities are calculated by multiplying the TOP-IDP propensity score with the difference in frequency between eukaryotes and one of the prokaryotes. Error bars represent the standard error for each amino acid. The amino acids are sorted according to their disorder propensity in the TOP-IDP scale.
