# Supplementary material for: Why do eukaryotic proteins contain more intrinsically disordered regions?
Source: PLoS Comput Biol. 2019 Jul 22;15(7):e1007186. doi: 10.1371/journal.pcbi.1007186 (PMC6675126; doi:10.1371/journal.pcbi.1007186)
Supplement: S4 Fig — Bacterial groups are red, eukaryotic dark green and archaeal blue. The amino acids are sorted by their one letter code. (PDF) [file pcbi.1007186.s011.pdf]

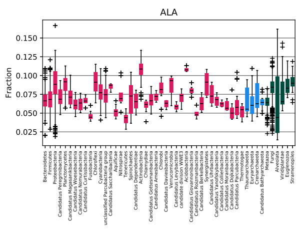

(a) Ala

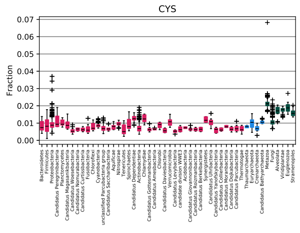

(b) Cys

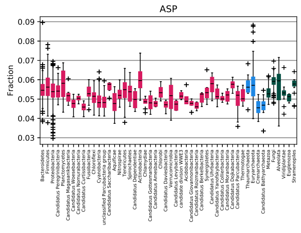

(c) Asp

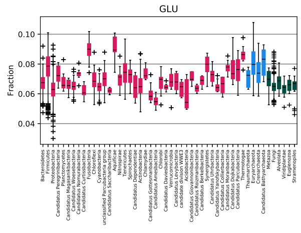

(d) Glu

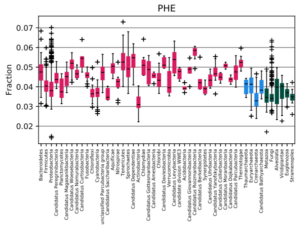

(e) Phe

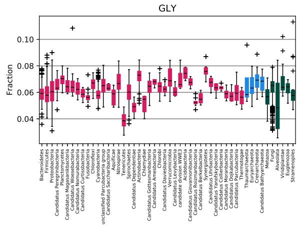

(f) Gly

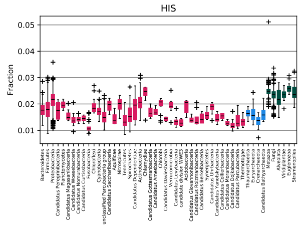

(g) His

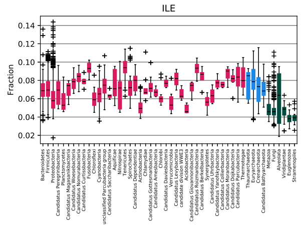

(h) Ile

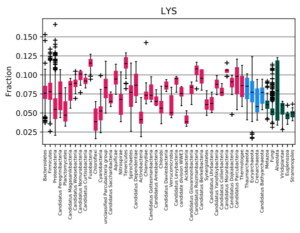

(i) Lys

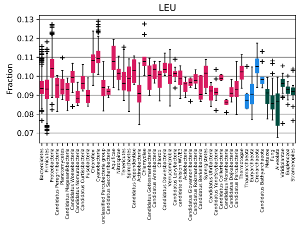

(j) Leu

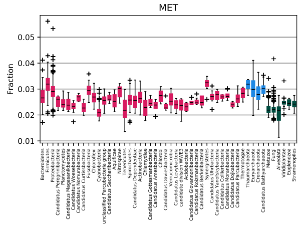

(k) Met

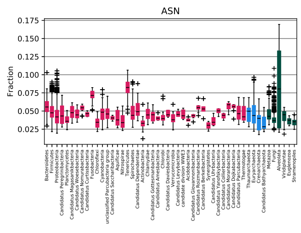

(l) Asn

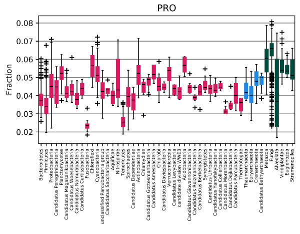

(m) Pro

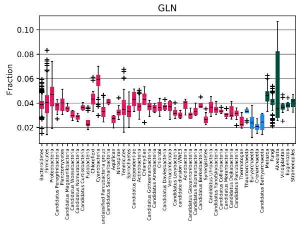

(n) Gln

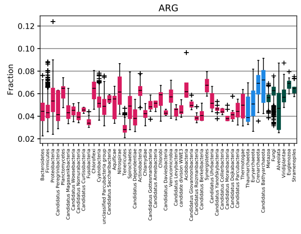

(o) Arg

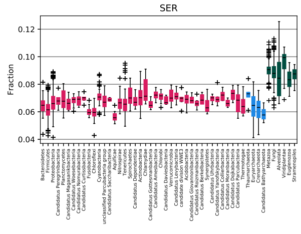

(p) Ser

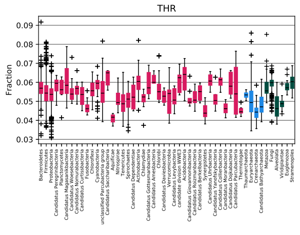

(q) Thr

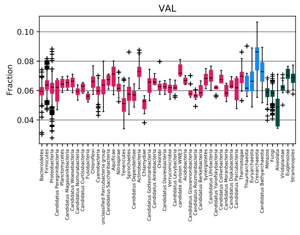

(r) Val

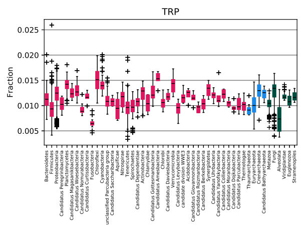

(s) Trp

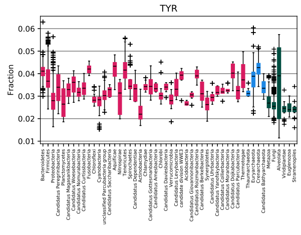

(t) Tyr

Figure S4. Frequency of amino acids in linker regions grouped by phylum. Bacterial groups are red, eukaryotic dark green and archaeal blue. The amino acids are sorted by their one letter code.
