# Supplementary material for: Why do eukaryotic proteins contain more intrinsically disordered regions?
Source: PLoS Comput Biol. 2019 Jul 22;15(7):e1007186. doi: 10.1371/journal.pcbi.1007186 (PMC6675126; doi:10.1371/journal.pcbi.1007186)
Supplement: S5 Fig — Red is bacteria, blue archaea and dark green eukaryota. Only the genomes that remained after filtering are included here. (PDF) [file pcbi.1007186.s012.pdf]

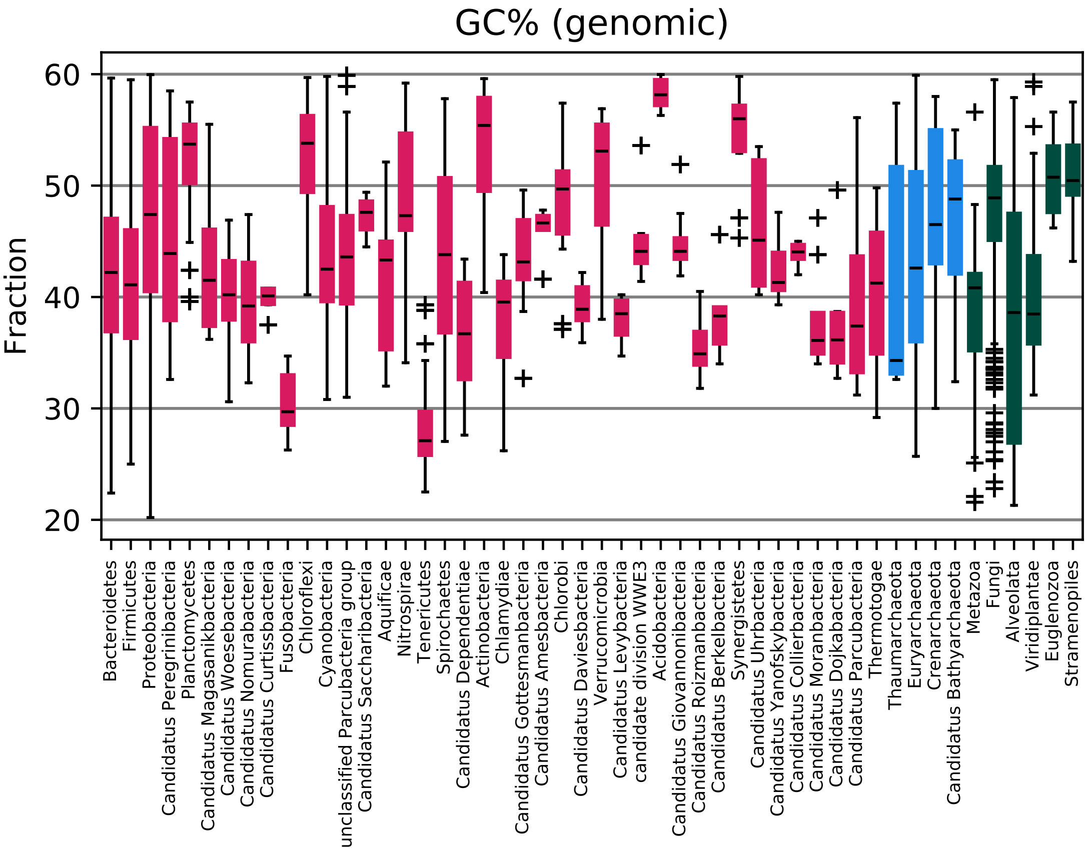

Figure S5. Distribution of genomic GC content for different phylogenetic groups. Red is bacteria, blue archaea and dark green eukaryota. Only the genomes that remained after filtering are included here.
