# Supplementary material for: Why do eukaryotic proteins contain more intrinsically disordered regions?
Source: PLoS Comput Biol. 2019 Jul 22;15(7):e1007186. doi: 10.1371/journal.pcbi.1007186 (PMC6675126; doi:10.1371/journal.pcbi.1007186)
Supplement: S6 Fig — (PDF) [file pcbi.1007186.s013.pdf]

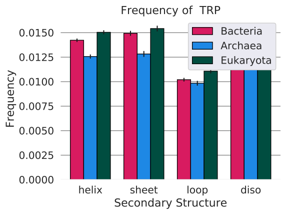

(a) Trp

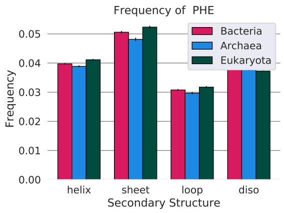

(b) Phe

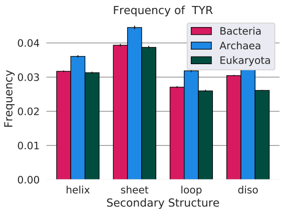

(c) Tyr

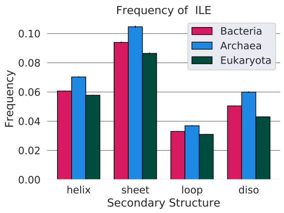

(d) Ile

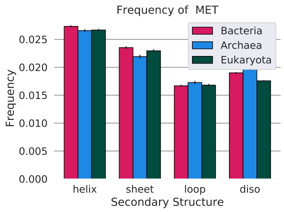

(e) Met

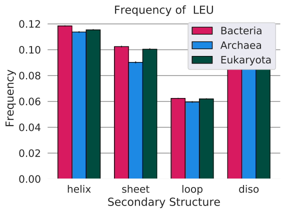

(f) Leu

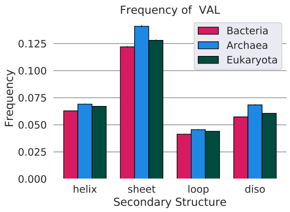

(g) Val

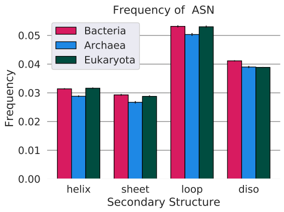

(h) Asn

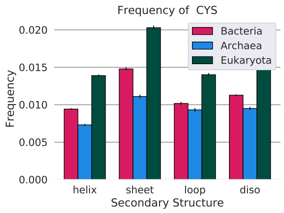

(i) Cys

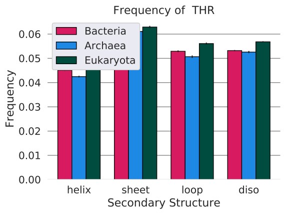

(j) Thr

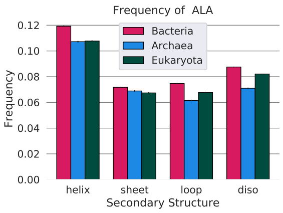

(k) Ala

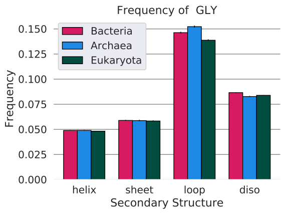

(l) Gly

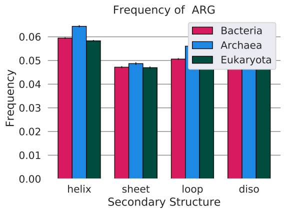

(m) Arg

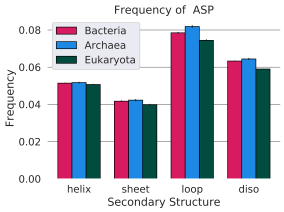

(n) Asp

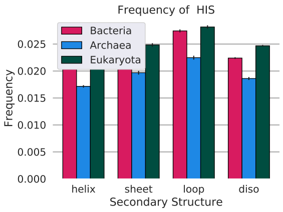

(o) His

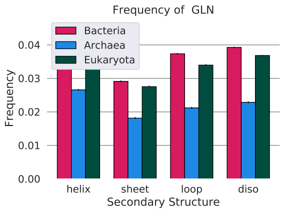

(p) Gln

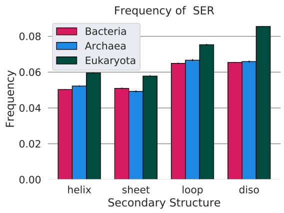

(q) Ser

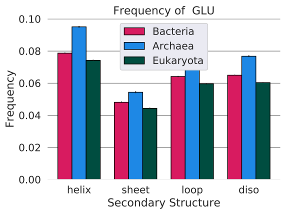

(r) Glu

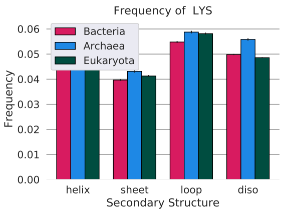

(s) Lys

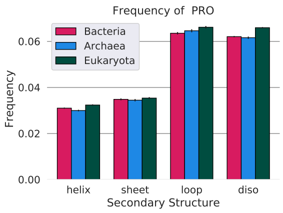

(t) Pro

Figure S6. Frequency of amino acids in different secondary structures in bacterial and eukaryotic proteins.
